# Targeting the Oxytocin Receptor for Breast Cancer Management: A Niche for Peptide Tracers

**DOI:** 10.1021/acs.jmedchem.3c01089

**Published:** 2024-01-18

**Authors:** Predrag Kalaba, Cristina Sanchez de la Rosa, Andreas Möller, Paul F. Alewood, Markus Muttenthaler

**Affiliations:** † Institute of Biological Chemistry, Faculty of Chemistry, University of Vienna, 1090 Vienna, Austria; ‡ Institute for Molecular Bioscience, The University of Queensland, Brisbane, Queensland 4072, Australia; § QIMR Berghofer Medical Research Institute, Brisbane, Queensland 4006, Australia; ∥ The Chinese University of Hong Kong, Hong Kong SAR 999077, China

## Abstract

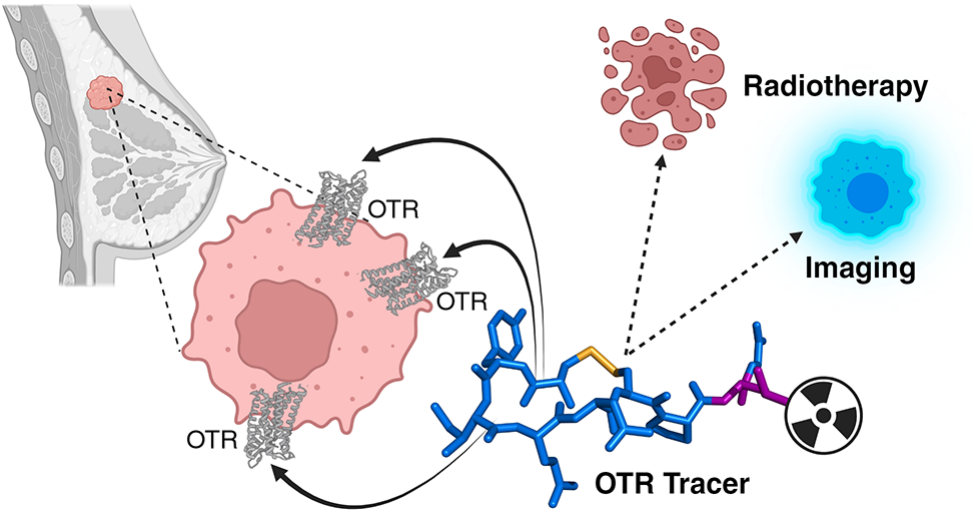

Breast cancer is a leading cause of death in women, and its management highly depends on early disease diagnosis and monitoring. This remains challenging due to breast cancer’s heterogeneity and a scarcity of specific biomarkers that could predict responses to therapy and enable personalized treatment. This Perspective describes the diagnostic landscape for breast cancer management, molecular strategies targeting receptors overexpressed in tumors, the theranostic potential of the oxytocin receptor (OTR) as an emerging breast cancer target, and the development of OTR-specific optical and nuclear tracers to study, visualize, and treat tumors. A special focus is on the chemistry and pharmacology underpinning OTR tracer development, preclinical *in vitro* and *in vivo* studies, challenges, and future directions. The use of peptide-based tracers targeting upregulated receptors in cancer is a highly promising strategy complementing current diagnostics and therapies and providing new opportunities to improve cancer management and patient survival.

## Significance

Receptors overexpressed in tumors but not healthy cells are promising targets for theranostics. The peptide hormone oxytocin receptor (OTR) is one such emerging target in breast cancer. Peptide-based optical and nuclear tracers are being developed that target OTR in breast cancer to validate OTR’s role and theranostic potential in breast cancer as well as to develop more effective, safer, and more personalized treatment options.

## 1 Breast Cancer—A Heterogeneous Disease

**Figure 1:**
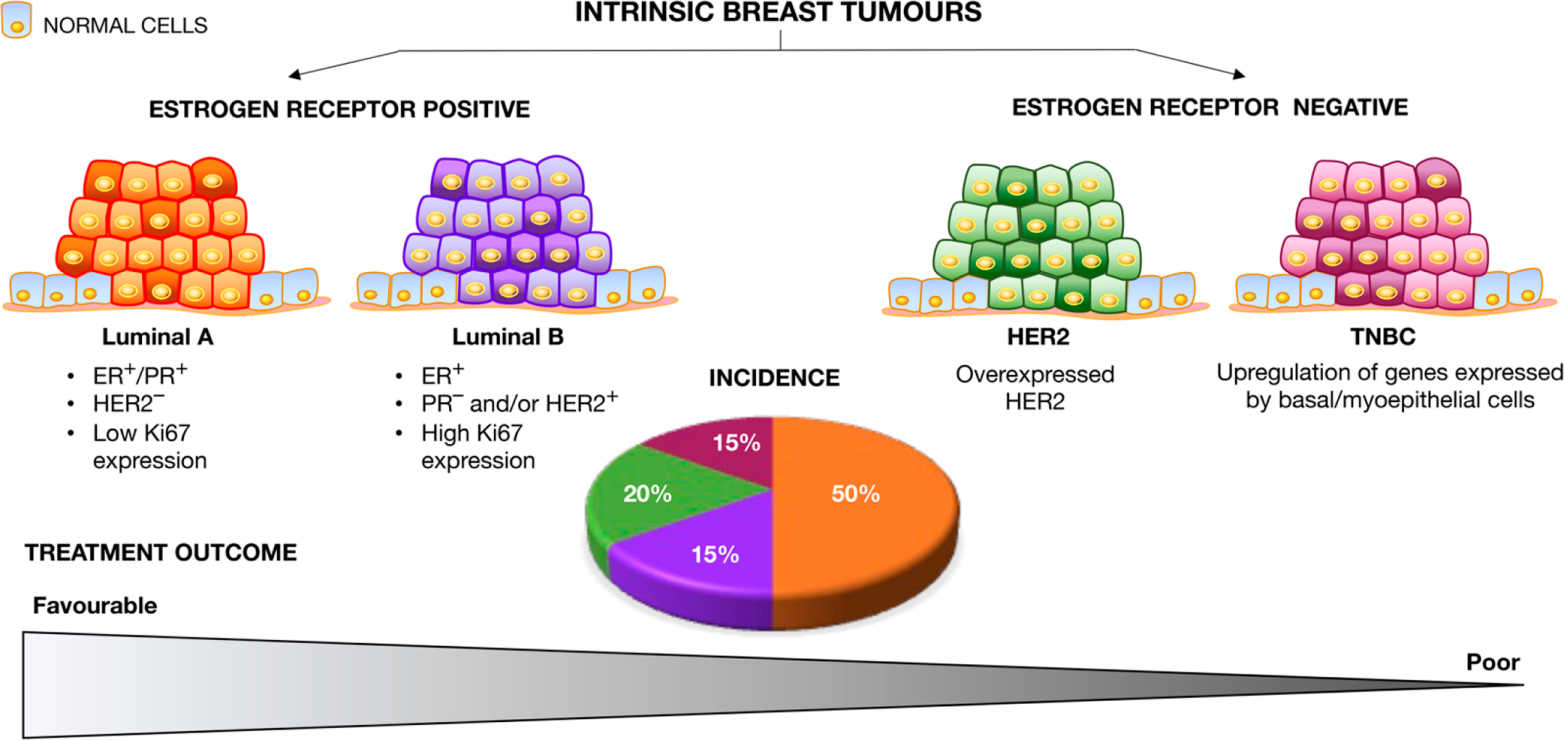
**Intrinsic subtypes of breast cancer.** They are represented as an accumulation of tumor cells in orange (luminal A), purple (luminal B), green (HER2), and dark pink (TNBC). The average incidence is presented in the pie chart, and the relative treatment outcome prognosis for the different tumor subtypes is shown in the bottom triangle (data on molecular profiles adopted from refs (22−24)). Ki67 is a nuclear protein associated with cell proliferation. A high fraction of Ki67-positive tumor cells suggests a high proliferation rate and is often indicative of more aggressive tumors.

Breast cancer is the second most common type of cancer diagnosed in women after non-melanoma skin cancers.^1^ The global cancer statistics reported 2.3 million newly diagnosed breast cancer cases and 684,996 deaths (responsible for 15.5% of total mortalities in females) in 2020.^2,3^ Despite having a favorable prognosis,^4^ breast cancer remains the leading cause of death in women worldwide.^2^ Efficient diagnosis and classification of breast cancer subtypes facilitate the management of the disease, but the heterogeneity of the malignant cells present in the mammary epithelial tissue often hinders accurate diagnosis.^5^ Current breast cancer diagnosis relies primarily on the assessment of molecular markers, such as the estrogen receptor (ER), progesterone receptor (PR), human epidermal growth factor receptor 2 (HER2),^6^ and Ki67, a cancer antigen used as a marker for cell proliferation and now an accepted prognostic factor to differentiate between ER-positive (ER^+^) tumor subtypes (Figure 1).^7,8^ Based on the expression of hormonal receptors, breast tumors can be classified into the following four subtypes: luminal A, luminal B, HER2-positive (HER2^+^), and triple-negative breast cancer (TNBC).^9^ Luminal A tumors have the highest incidence (∼50%) among women but also have the best therapeutic outcome; by contrast, luminal B tumors have a lower incidence (∼15%) but worse prognosis.^9^ ER-negative (ER^–^) breast tumors overexpressing HER2 (incidence of ∼20%) initially had poor therapy outcomes,^9^ which have improved in recent years due to the introduction of combination therapies.^10^ FDA-approved anti-HER2 agents include monoclonal antibodies Pertuzumab, Trastuzumab, and Ado-Trastuzumab emtansine (T-DM1);^11^ tyrosine kinase inhibitors Lapatinib (TYKERB),^11,12^ Neratinib (Nerlynx),^12^ and Tucatinib (Tukysa);^12^ and the antibody–drug conjugate fam-trastuzumab deruxtecan-nxki (DS-8201a, T-DXd, ENHERTU).^13^ Finally, tumors that are negative for the biomarkers mentioned above (ER^–^, PR^–^, HER2^–^) fall in the TNBC category. TNBC includes basal (neoplastic cells constitutively expressing markers, such as cytokeratins or EGFR)^14^ and non-basal tumors,^15^ with most TNBCs expressing basal markers.^14^ TNBC patients have poor overall survival due to the tumors’ aggressiveness and increased risks of relapse^16,17^ and have an incidence rate of ∼15%.^9,18^ Pembrolizumab, a humanized monoclonal anti-programmed cell death protein 1 (PD1) antibody, is used as a neoadjuvant and adjuvant for treating patients with high-risk early-stage TNBC.^19,20^

According to the “5-year relative survival percentage” reported by the U.S. National Cancer Institute, the highest survival pattern was observed in women with luminal A subtype (94.4%), followed by the luminal B subtype (90.7%), HER2 subtype (84.8%), and finally TNBC (77.1%).^9^ Although mortality has decreased due to early detection and increasing therapeutic options, almost 30% of patients diagnosed with early stages of breast cancer still develop recurrent or metastatic diseases,^9^ with 5-year survival rates in those patients of only 27%.^21^

## 2 Diagnostic Tools for Breast Cancer Management

In most countries, manual breast palpation is the first screening method to detect breast tumors,^25^ followed by visualizing abnormalities through different breast imaging techniques. An overview of current technologies for breast cancer detection is presented in Table 1, including their advantages and limitations.

**Table 1:**
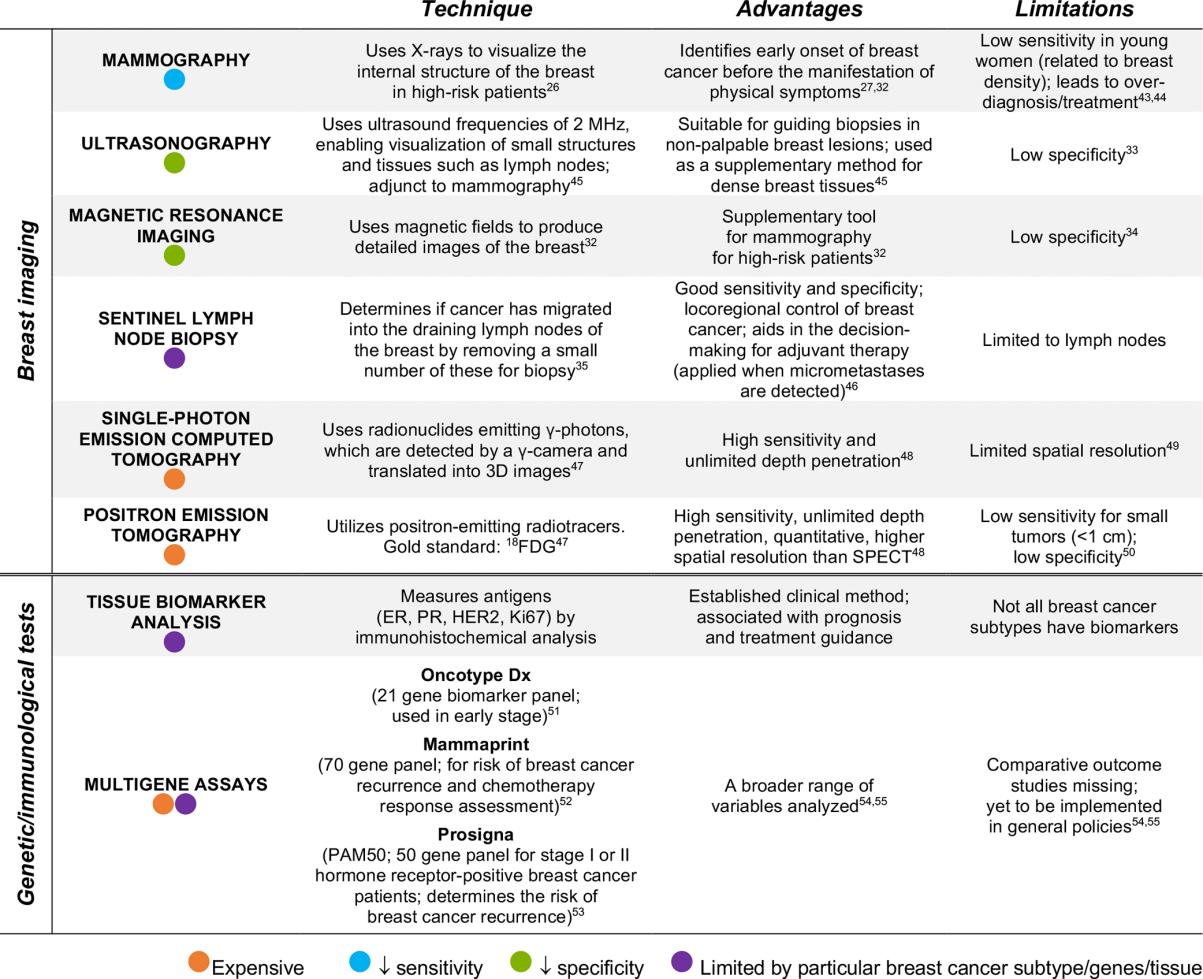
Current Breast Cancer Detection and Diagnostic Methods, Including Their Advantages and Limitations^a^

a A visual overview of the three main limitations is represented with colored circles. “Sensitivity”, in a clinical context, refers to the amount of correctly diagnosed patients (i.e., true positives), as opposed to the term “specificity”, which alludes to the number of healthy people correctly diagnosed as not having the disease (i.e., true negatives).^29^ Stage I, tumor less than 2 cm; stage II, tumor 2–5 cm.

Mammography utilizes a low dose of X-rays to visualize the internal structure of the breast.^26−29^ It is widely used to identify the early onset of breast cancer before the manifestation of physical symptoms.^26,27^ It has, however, low sensitivity in young women due to higher breast tissue density and higher tumor growth rate than in older women.^28^ Even though mammography is the gold standard used for breast cancer screening,^30^ other techniques such as ultrasonography^31^ and magnetic resonance imaging (MRI)^32^ are used to identify tumors that are not detectable in mammograms, as well as to determine tumor size more accurately. Each of these modalities has advantages and limitations, mostly due to their low specificity (i.e., high rate of false positives).^33,34^ Other techniques, such as sentinel lymph node biopsy (SLNB)^35^ or molecular profiling,^36^ aid in categorizing the stage of breast tumors.

Nuclear imaging techniques such as single-photon emission computed tomography (SPECT) and positron emission tomography (PET) are used as auxiliary imaging tools to better characterize breast cancer. SPECT and PET can detect tumors using radiotracers (small molecules, peptides, antibodies, affibodies, or nanobodies labeled with radioisotopes) directed to receptors, transporters, or enzymes overexpressed in cancer cells.^37−41^ The clinical use of SPECT and PET remains limited due to the high costs (especially in the case of PET tracers that have short half-lives and require in-house cyclotron production) and scarcity of radiotracers. However, considering the ongoing technological advances, we expect to see a broader utilization of these technologies, particularly due to their high sensitivity and accuracy.^42^

**Figure 2:**
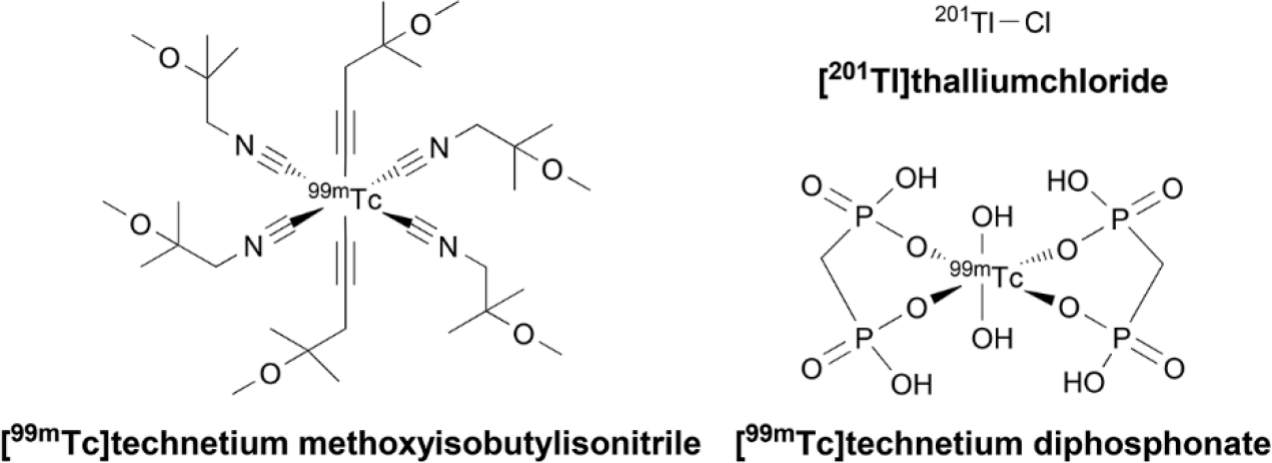
**Radiopharmaceuticals used in SPECT imaging.**

SPECT uses radiotracers that emit γ-rays captured with a γ camera to acquire multiple 2D projections from different angles to produce a full 3D body image.^56^ SPECT-CT permits accurate 3D localization of primary and/or metastatic tumors, yet with a limited spatial resolution (∼10 mm).^49^ Typical radiopharmaceuticals used in SPECT imaging of breast cancer include [^201^Tl]thalluimchloride, [^99m^Tc]technetium methoxyisobutylisonitrile ([^99m^Tc]Tc-MIBI, [^99m^Tc]Tc-sestamibi), and [^99m^Tc]technetium diphosphonates (Figure 2).^57^ As an example, SPECT-CT with [^99m^Tc]Tc-MIBI is used for finding proliferative tumoral tissue around a breast implant, in the remaining breast parenchyma, or on the chest wall after surgery.^58^

PET imaging is based on detecting annihilation photons produced by the disintegration of positron-emitting radiotracers.^59^ The most commonly used radiotracer for the visualization of tumor distribution is [^18^F]fluorodeoxyglucose ([^18^F]FDG), a radiolabeled glucose analogue that cancer cells absorb in greater amounts than normal cells due to their increased metabolic activity.^47^ The different pharmacokinetic (PK) and pharmacodynamic (PD) rates are measured throughout the body with high spatial resolution, allowing the visualization of cancer metastases far away from the breast. A limitation of PET is its deficient detection rate for non-invasive breast cancers and small breast carcinomas.^60,61^ Sensitivity of PET highly depends on tumor size,^50^ varying from 95% for tumors larger than 1 cm to 25% for tumors smaller than 1 cm (resolution limit in modern clinical PET scanners is 4 mm).^62^ This fact renders the detection of early-stage breast cancer (stage I, tumors 1–19 mm) challenging.^60,63^ Moreover, the variability of glucose uptake in primary breast tumors differs in terms of tissue integrity and vascular density, which can result in false negatives that are difficult to differentiate from real signals.^64^

Both SPECT and PET can assess the presence and extent of disease as well as provide unique information about tumor biological characteristics, such as the rate of proliferation and metabolic activity.^57^ These methods are important and powerful techniques to complement traditional imaging modalities in breast cancer diagnosis and monitoring. The therapeutic potential of these techniques is leveraged when targeted approaches are employed, directing a radiopharmaceutical to the tissue of interest and differentiating it from healthy tissue.

## 3 Receptor-Targeted Approaches

The discovery that several cell surface receptors are overexpressed in tumor tissues compared to healthy tissue enables tumor-targeting strategies.^65^ Targeting entities include small molecules, peptides, antibodies, or antibody fragments, each having their advantages and limitations. Small molecules, for example, offer good bioavailability, stability, and tumor tissue penetration but are often not target-specific due to their small size and limited chirality.^66^ By contrast, antibodies provide high target specificity and affinity but have to be injected and have poor tumor penetration due to their large size.^67^ Peptide ligands display a good balance in biochemical properties between small molecules and antibodies, having higher tissue penetration than antibodies and better target specificity and affinity than small molecules.^67−70^ While some peptides have short half-lives, it is relatively easy to tune their metabolism and clearance rate, e.g., using fatty acid modifications for serum albumin binding.^71^ Moreover, peptides can be equipped easily with reporter tags compatible with optical (fluorescent tracers) or nuclear (radiotracers) imaging.^68,72^ Such peptide tracers, therefore, hold promising potential as diagnostic and therapeutic tools in oncology.^72,73^

### 3.1 Tracers for Optical Imaging

Optical imaging tracers are typically equipped with fluorophores to visualize membrane receptors expressed in cancer cells. While fluorescent ligands can be used *in vitro*, tissue autofluorescence needs to be considered for *in vivo* imaging since mammalian tissues are opaque to light in the visible spectrum (400–700 nm).^74^ In these cases, near-infrared (NIR) fluorophores can be used that function in wavelength regions above 700 nm.^75^ NIR fluorophores reduce light-scattering effects, enabling better tissue penetration (>1 cm). NIR imaging is applied in superficial tumor detection, integrated as part of endoscopies and open-surgery procedures, which assists surgeons in removing cancerous tissue;^72,76^ indocyanine green (ICG) and methylene blue (MB) are so far the only two NIR fluorophores approved by the FDA.^75,77^ NIR peptide tracers have been developed targeting overexpressed EGFR in glioblastomas^78^ and the integrin αvβ3 receptor expressed on sprouting tumor vasculatures.^79^ Most NIR tracer research is still preclinical, focusing on improving optical properties and toxicity profiles,^80,81^ with the exception of BLZ-100 (Tozuleristide, Tumor Paint; chlorotoxin peptide conjugated to ICG), which is expected to receive FDA approval for its use in visualizing pediatric brain cancer cells during tumor-removing surgery and has also been applied to breast cancer.^82,83^

### 3.2 Tracers for Nuclear Imaging

**Figure 3:**
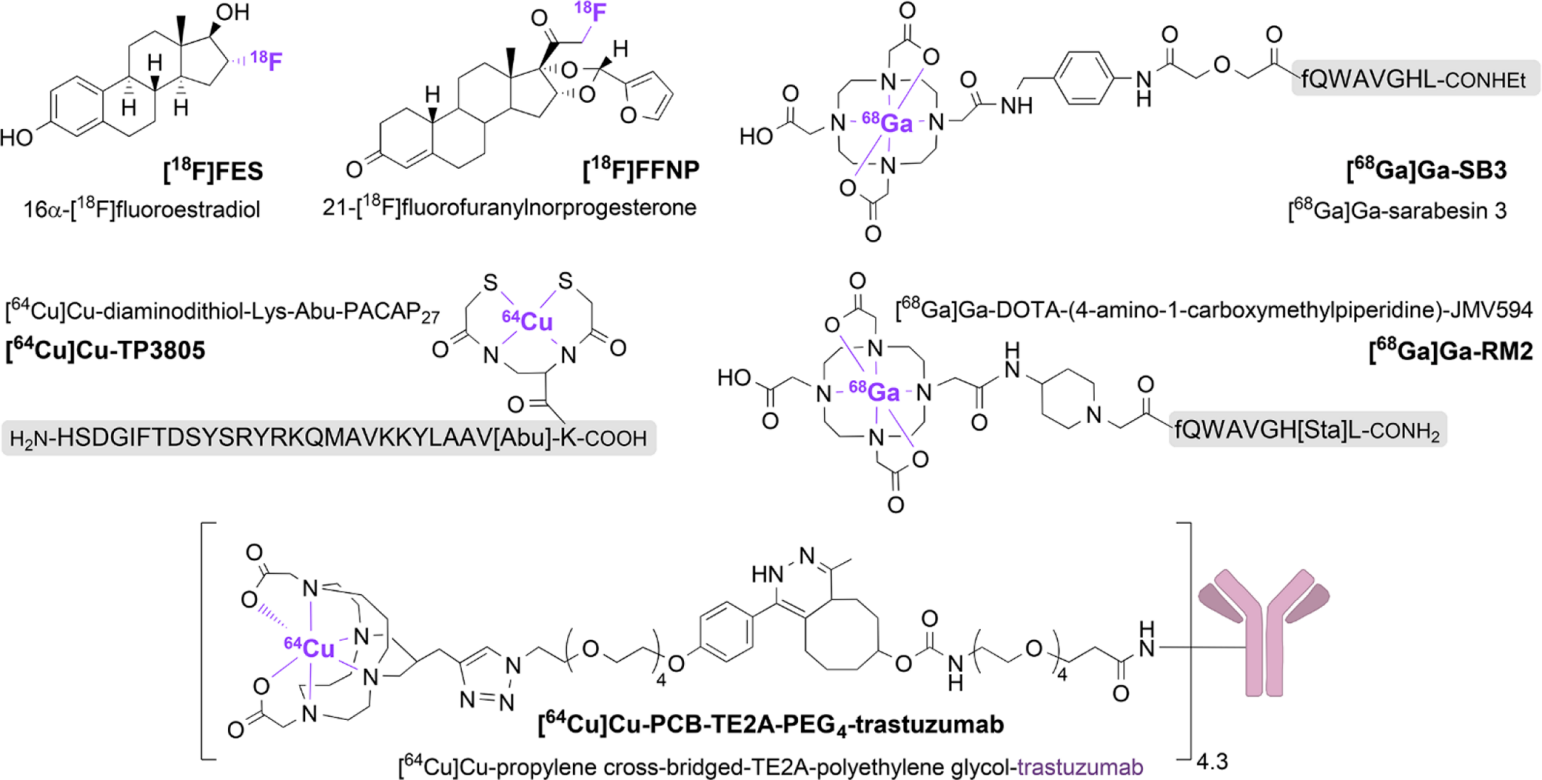
**Tracers used for nuclear imaging of different breast cancer types.** Ligands, linkers, and chelators are colored in black, radionuclides in purple, and antibodies in mauve. Abu, 4-aminobutyric acid; Sta, statine ((3*S*,4*S*)-4-amino-3-hydroxy-6-methylheptanoic acid).

Nuclear imaging tracers use radioisotopes to visualize cancer cells. Particularly SPECT and PET techniques are employed to provide 3D positional images of targeted tumors in the body;^47^ this relates to whole-body as well as focused breast imaging, although the latter offers better diagnostic accuracy.^84,85^ Radiotracers can be used in the clinic for disease scanning, tumor characterization (staging), and treatment response monitoring.^37^ The design and synthesis of radiotracers for breast cancer (Figure 3) were initially based on the labeling of identified biomarkers (ER, PR, and HER2). The [^18^F]FES PET radiotracer has been used in ER^+^ tumors for preclinical evaluation of a therapeutic response and clinical visualization of breast cancer with an average of 90% specificity and 85% sensitivity.^86−92^ In the case of PR, [^18^F]FFNP was used in a clinical study, identifying ∼94% of PR^+^ breast tumors using PET.^93^ To visualize HER2^+^ breast tumors, both PET and SPECT radiopharmaceuticals based on the monoclonal antibody trastuzumab were tested,^14−18,94^ and the efficacy of ^64^Cu-labeled trastuzumab to detect HER2-positive breast cancer was confirmed.^95^

Less studied targets include the gastrin-releasing peptide receptor (GRPR) and vasoactive intestinal polypeptide receptor 1 (VIP-R1), yet their expression in breast cancer is documented (GRPR^96−99^ and VIP-R1^100,101^), and they were investigated clinically for the detection of breast malignancies.^102−104^ Radiotracers used for the detection of GRPR were [^68^Ga]Ga-SB3, achieving 50% tumor detection (*n* = 8),^102^ and [^68^Ga]Ga-RM2, reaching 72% breast cancer visualization (*n* = 18),^103^ both GRPR peptide antagonists. In a clinical study to detect VIP-R1, radiotracer [^64^Cu]Cu-TP3805 was used, achieving 100% detection of breast tumors (*n* = 20).^104^

Expression of target receptors often depends on breast cancer subtype and might be influenced by factors such as treatment or disease progression.^37^ TNBCs currently lack validated target receptors or biomarkers,^105^ and targeting metastatic disease is challenging, with a poor 5-year survival (<30%).^106^ It remains therefore important to identify and validate new receptor targets for breast cancer management, especially for TNBC. The peptide hormone oxytocin receptor (OTR) is such a new target.^107,108^

## 4 The Oxytocin Receptor: An Emerging Target for Breast Cancer Management

**Figure 4:**
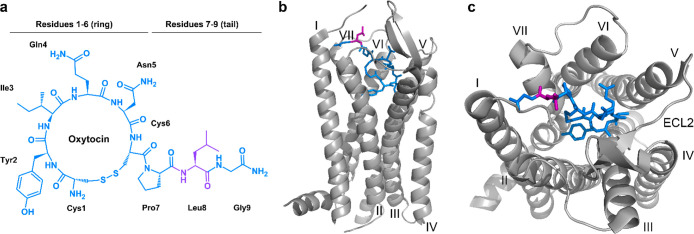
**OT chemical structure and the OT-OTR complex.** (a) Molecular structure of OT (blue) and the recommended labeling position 8 indicated in purple. (b) Side view of OT bound to OTR. (c) Top view of OT bound to OTR, as determined via single-particle cryo-electron microscopy of OTR in complex with OT (adapted from ref (112)). For a detailed map of molecular interactions, please refer to Waltenspühl et al.^112^

OTR belongs to the class I family (rhodopsin-like) of G protein-coupled receptors (GPCRs) and is activated by its endogenous peptide hormone OT.^109^ OT is a nonapeptide comprising a six-residue macrocycle linked by a disulfide bond between positions 1 and 6 and a three-residue tail with a *C*-terminal amide (Figure 4a). OTR’s structure has recently been resolved via X-ray diffraction, with antagonist retosiban bound to an inactive conformation,^110^ and via cryo-EM, with OT bound to an active conformation (Figure 4b,c).^111,112^ OTR can be upregulated to play important roles in the reproductive, cardiovascular, endocrine, and central nervous systems.^109^ OTR becomes upregulated in the female breast during pregnancy when the mammary glands develop in preparation for breastfeeding.^107,109^ During breastfeeding, OT is synthesized in the magnocellular neurons of the hypothalamus, transported to the posterior pituitary (neurohypophysis),^109,113^ from which it is released into the bloodstream to bind to OTR in the mammary glands, inducing contractions of the myoepithelial cells resulting in milk ejection.^114^ OTR is also expressed in breast tumors,^107,115^ as well as leiomyoma,^116^ neuroblastoma and glioma,^117^ adenocarcinoma of the endometrium,^118^ ovarian carcinoma,^119^ prostate cancer,^120−122^ small-cell lung carcinoma,^123^ trophoblast,^124^ choriocarcinoma,^124^ and osteosarcoma.^125,126^ Peptide radiotracers have been developed successfully targeting OTR in breast malignancies (mouse models),^127,128^ supporting sufficient receptor density in tumors for diagnosis and treatment. OTR mRNA was detected in up to 95% of human breast cancer cell lines (*n* = 60)^129−134^ and tissues (*n* = 57).^130,131^ OTR was detected at the protein level in 91% of such tissue samples,^130,133^ although immunohistochemistry results need to be considered cautiously due to potential problems related to OTR antibody specificity and lack of appropriate controls. Proliferation assays using human epithelial triple-negative MDA-MB-231 and ER^+^ MCF7^135^ breast cancer cell lines demonstrated increasing antiproliferative effects when OT was administered in a dose-dependent manner (1–100 nM), consistent with previous data.^136^

Administration of OT and OT analogues in different rodent breast cancer models reduced breast tumors substantially (44–82%).^107,115,137−139^ For example, OT or atosiban (biased OTR ligand) was administered via osmotic pumps into xenograft models of BALB/c mice (*n* = 43) and Fisher rats (*n* = 22) bearing mammary carcinomas TS/A and D-R3230AC, respectively, for 14 days.^115^ This resulted in up to 72% tumor reduction in animals treated with OT or atosiban compared to controls. Tumor reduction by atosiban was unexpected since it was thought to be an OTR antagonist; further studies, however, demonstrated atosiban to be a biased ligand, selectively activating G_i_ signaling while blocking G_q_ signaling.^140^ In another study that used BALB/c mice bearing MC4-L2 mouse mammary adenocarcinomas, treatment with OT for 42 days reduced tumor growth rate and tumor size (∼82%).^137^ Treatment with atosiban was also studied; however, no significant changes in tumor growth or size were observed. Using the same xenograft system, BALB/c mice bearing MC4-L2 mouse mammary adenocarcinomas (*n* = 56), a 44% tumor reduction upon OT treatment and a 12% increase in tumor volume with atosiban were observed.^138^ In another study, OT was administered for 21 days in BALB/C mice bearing mammary carcinomas TS/A, which led to a tumor reduction of ∼50%.^107,139^ While tumor reduction upon OT treatment seems fairly consistent among studies, atosiban displayed varying effects (reduction/proliferation of tumors or no effect in tumor growth), warranting more systematic studies. OT also seems to be released upon physical activity, acting protectively against breast cancer:^141^ mice bearing breast tumors were assigned to a treadmill for 5 days, increasing OT concentration in plasma. Since an endogenous OT increase was also observed in parallel to tumor growth inhibition effects, a different group of animals was externally administered with OT (no exercise), confirming OT-dependent tumor reduction (tumor volume 42 days after tumor transplantation, tumor + exercise training, and tumor + OT was 1.10, 0.81, and 0.56 cm^3^, respectively).^138^ All of the used breast cancer cell lines express OTR. The MCF7 cell line originates from a patient with metastatic breast cancer and is the most studied human breast cancer cell line in the world,^142^ while TS/A originated from a spontaneous mammary tumor of an inbred BALB/c female mouse exhibiting features typical of human breast cancer.^143^ MC4-L2 xenograft models are experimental models of mammary carcinogenesis in which the administration of medroxyprogesterone acetate to female BALB/c mice induces progestin-dependent ductal metastatic mammary tumors with high levels of both ER and PR.^144^ Despite the overall beneficial effects of OT in these animal models, more studies are required to investigate the effects of OT and optimized/selective OTR drug candidates on human xenograft models across a variety of different subtypes. OT itself is considered a poor drug candidate due to its short circulation half-life and activity at closely related vasopressin receptors (VPRs).

In a clinical study, endogenous OT increased up to 9-fold in the peripheral blood from tumor tissue of breast cancer patients (*n* = 40) compared to contralateral (healthy) breast; this effect increased in the advanced stages of the disease (*p* ≤ 0.004).^145^ Interestingly, OTR gene and protein expression were up to 11-fold downregulated.^145^

The relationship between breastfeeding and breast cancer has been studied since the 1950s.^146^ The consensus is that breastfeeding reduces the risk of developing breast cancer.^147−155^ The most comprehensive study with the highest statistical significance to date was published in 2002, a worldwide collaborative analysis comprising 47 studies which included population-based, case-control, and follow-up analyses in parous women of different ages and ethnic origins in developed and developing countries, and with a wide range of reproductive and breastfeeding patterns.^148^ The relative risk of breast cancer development in this study was reduced by 4.3% per year in women who breastfed for at least 12 months. Another study with women in Sri Lanka (*n* = 19,755) demonstrated a striking reduction of 87–94% in the risk of developing breast cancer among women who breastfed for more extended periods (24–47 months).^155^ The exact mechanisms underpinning these protective effects require further investigation; however, evidence points to an involvement of the OT/OTR signaling system.

The exact expression profiles of OTR in breast tumors of different subtypes and stages remain an active research question; however, OTR-specific nuclear imaging tracers accumulated in breast malignancies *in vivo*, suggesting that OTR expression is sufficient for tumor-specific targeting, at least in the rodent models employed.^127,128,156^ More systematic studies investigating OTR expression across a large panel of breast cancer patient samples will be critical in revealing cancer subtypes that display robust OTR overexpression to support tumor imaging and targeted radiotherapy.

## 5 Oxytocin Receptor Tracer Development

**Figure 5:**
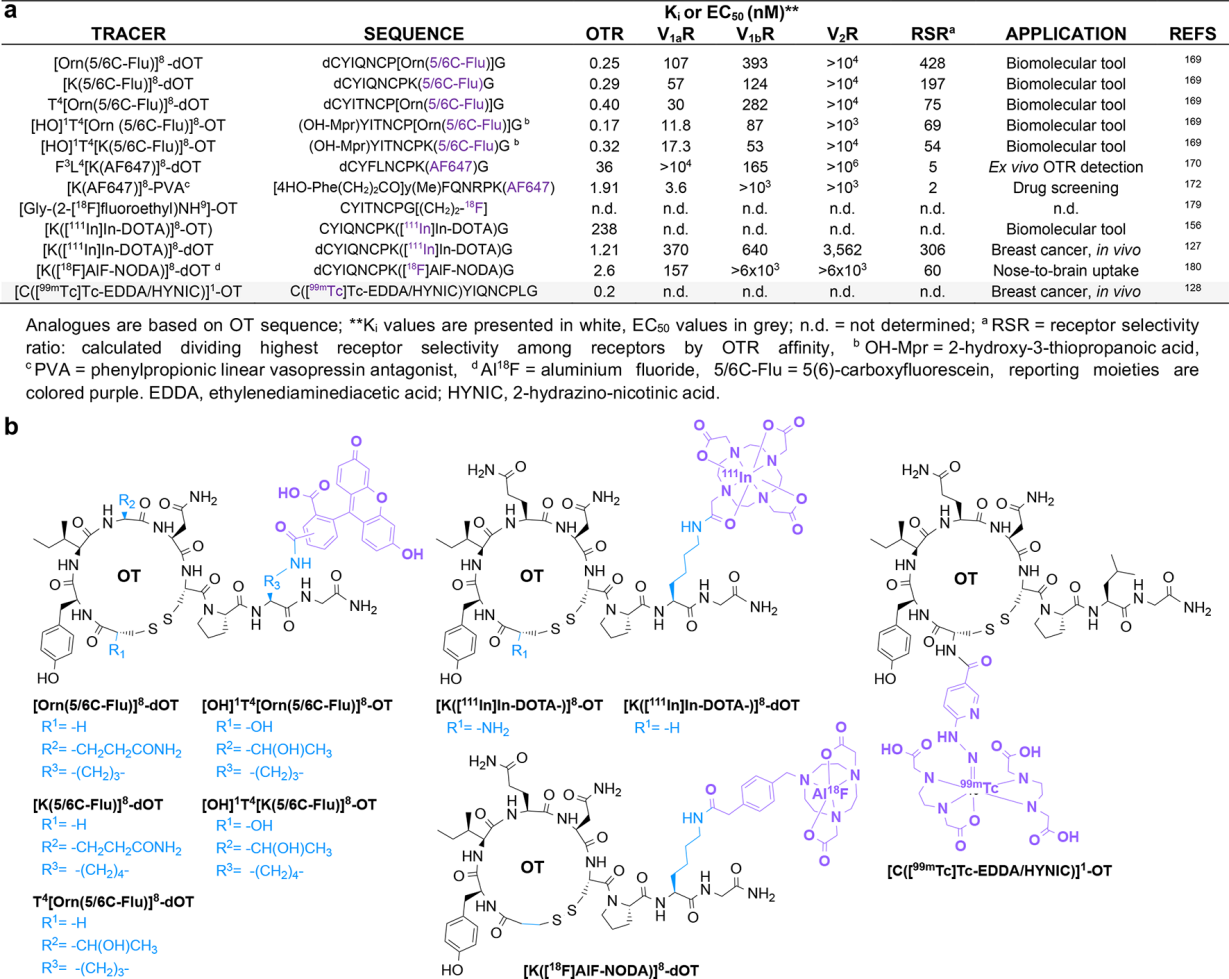
**Overview of developed OTR tracers with information on OTR and VPR pharmacology, OTR selectivity, application, and chemical structure.** (a) OTR-targeting fluorescent tracers and radiotracers with their sequence information, the OTR and VPR pharmacology, the OTR selectivity ratio (RSR), and the application information. (b) Chemical structures of all listed OTR tracers. Residues that differ from endogenous OT are highlighted in blue and labeling groups (fluorophores, radionuclides, and metal chelators) in purple.

A major bottleneck in OTR ligand development is OTR’s similarity to the closely related VPRs, V_1a_R, V_1b_R, and V_2_R.^157−159^ Considerable efforts have been invested in structure–activity relationship (SAR) studies and medicinal chemistry approaches, with thousands of ligands synthesized and pharmacologically characterized.^157,158,160−164^ This resulted in several approved peptide drugs acting via OTR (e.g., OT, demoxytocin, atosiban, carbetocin); however, none of these ligands is OTR-selective,^157^ with selectivity defined as a 100-fold affinity preference for one receptor over the others.^165^Table 2 lists a range of agonists and antagonists with selectivity/preference for OTR. Models of ligand–receptor interactions originally suggested that the OT ring fits deeply into the transmembrane (TM) core, interacting with a cluster of residues located in TM3, TM5, and TM6, whereas the tail interacts with the upper part of the first TM helix and the second extracellular domain of the receptor (Figure 4b,c).^166−168^ Recently elucidated OT-bound OTR structure via single-particle cryo-EM revealed that all 9 amino acids of the OT participate in OTR binding with the cyclic part (residues 1–6) being deeply buried inside of the pocket while the C-terminal tripeptide (7–9) is situated toward extracellular loops (Figure 4b,c).^112^ Leu^8^ is oriented toward the extracellular space, explaining why position 8 is best suited for attaching fluorophores or other modifications in OT.^112^ Position 8 of OT (Figure 4a, purple) has been replaced or modified with several moieties without substantially affecting OTR binding or activation. Particularly replacement of Leu^8^ with lysine or ornithine provides a suitable handle for OT-like tracer production.^127,169−172^ This modification is often combined with removing the *N*-terminal amine, rendering the peptide less susceptible to aminopeptidases and more hydrophobic with a better binding pocket fit and enhanced potency.^173^ Several OTR tracers have been developed^169^ and are listed and depicted in Figure 5, along with their pharmacological profiles across OTR and VPRs and their applications.

**Table 2:**
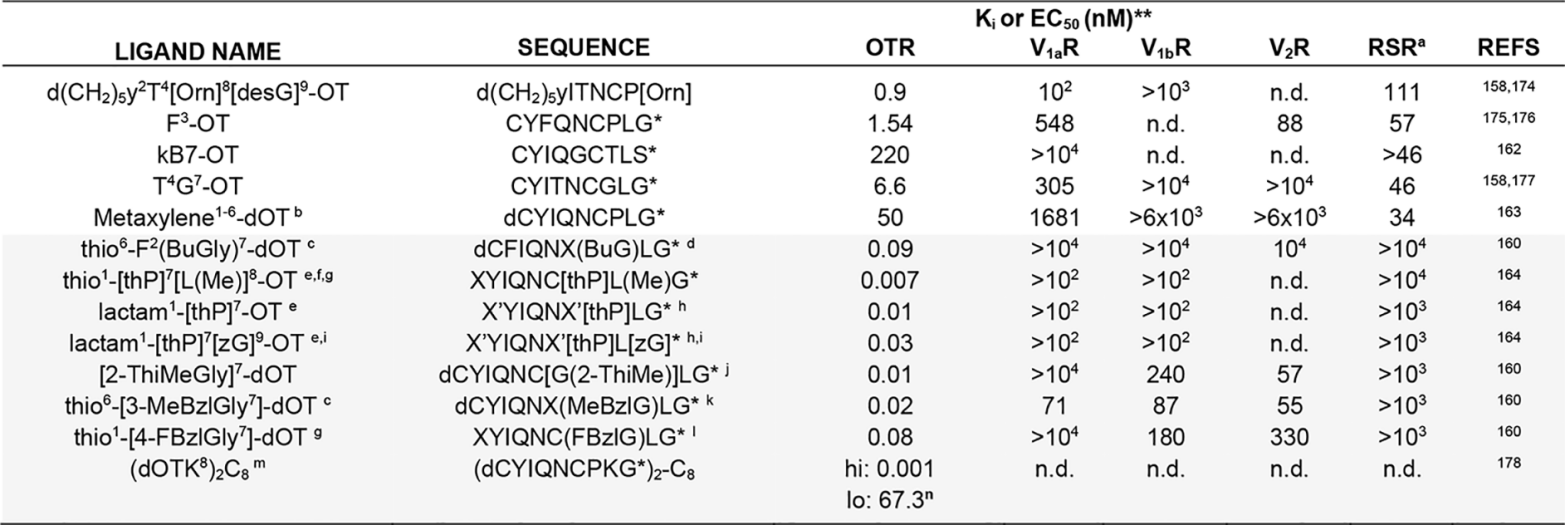
OT Analogues with OTR Preference or Selectivity over VPRs

a RSR, receptor selectivity ratio: calculated dividing highest receptor selectivity among receptors by OTR affinity.

b Disulfide bridge formed with substituted dibromoxylene (*m*-xylene bridged between residues 1 and 6).

c thio = S–S bridge was substituted for CH_2_–S, with CH_2_ in position 6.

d BuG, *N*-(*n*-butyl)glycine.

e thP = *trans*-4-hydroxyproline.

f L(Me) = γ-methylleucine.

g thio = S–S bridge was substituted for CH_2_–S, with CH_2_ in position 1.

h X′ amino acids used to form a lactam bridge.

i zG = azaglycine.

j G(2-ThiMe) = *N*-alkylated glycines with 2-thiophenylmethyl.

k MeBzlG = *N*-(3methylbenzyl)glycine.

l FBzlG = *N*-(4-fluorobenzyl)glycine.

m C_8_ = octanoic acid.

n Biphasic fitting (Hill coefficient for both phases was fixed to −1; “EC_50_ hi” refers to the high-affinity site and “EC_50_ lo” to the low-affinity site.^178^ All assay values were obtained from pharmacological testing on human isoforms of OTR, V_1a_R, V_1b_R, and V_2_R. *Analogues are based on OT sequence (indicates *C*-terminal amide). **K_i_ values are presented in white, EC_50_ values in gray. n.d. = not determined; “d” = for desamino (no N-terminal amine); d(CH_2_)_5_ = 1-[β-mercapto-β,β-cyclopentamethylene]propionic acid.

Some of these OTR radiotracers have been used to target breast cancer.^127,128^ ([K([^111^In]In-DOTA)]^8^-OT) displayed high affinity for OTR (MCF7 breast cancer cells) and tumor uptake (BALB/c mice bearing OTR^+^ TS/A tumor) with a tumor-to-blood ratio of 2.67.^156^ The desamino version of this tracer, [K([^111^In]In-DOTA)]^8^-dOT, was evaluated in a preclinical study aiming to determine the amount and specificity of the receptor-mediated uptake using an experimental model of OTR^+^ TS/A tumors growing in Balb/c mice.^127^ This biodistribution study demonstrated higher tracer uptake in tumors than in blood or liver (tumor/blood and tumor/liver uptake ratios were 7.58 and 1.42, respectively) but lower than in kidneys (tumor/kidney uptake ratio was 0.06), highlighting a rapid clearing process.^127^ OTR-specificity was confirmed by administering 50 μg of OT 30 min before radiotracer administration, resulting in a 3-fold-reduced tumor uptake.^127^ [K([^111^In]In-DOTA)]^8^-dOT internalized with OTR within 5 min, supporting tracer tumor accumulation, which could be beneficial for tumor imaging or targeted radiotherapy.^127^

[C([^99m^Tc]Tc-EDDA/HYNIC)]^1^-OT is another radiotracer developed to target OTR.^128^ Here, 2-hydrazinonicotinic acid (HYNIC) chelates technetium-99m, a γ-emitting radionuclide with a half-life of 6 h commonly used for SPECT.^181^ As such complexes may exist in numerous isomeric forms, adding ethylenediaminediacetic acid (EDDA) as co-ligand helps prepare complexes of higher stability and symmetry, resulting in fewer coordination isomers.^182^ Interestingly, the addition of the chelator HYNIC to the *N*-terminal amine of OT did not affect OTR binding,^183^ while introducing [^99m^Tc]Tc-EDDA/HYNIC to position 8 reduced binding affinity, as determined by a radioimmunocompetition assay (IC_50_ values of 0.2 and 1 nM, respectively). Both Lys^8^- and Cys^1^-labeled tracers were internalized in OTR-expressing MCF7 cells. *In vivo*, breast tumors were induced in athymic male mice by subcutaneous injection of MCF7 cells. From the two studied labeling positions, only [C([^99m^Tc]Tc-EDDA/HYNIC)]^1^-OT achieved tumor uptake, besides the typical non-specific uptake in kidneys and liver.^128^

## 6 OTR Tracers for Breast Cancer—Opportunities and Challenges

OTR radiotracers have shown promise in breast cancer mouse models with OTR and tumor-specific uptake.^127,128,156^ Rational design to advance OTR tracer development is feasible, particularly considering the many SAR studies with OT analogues along with the well-established OTR/VPR pharmacology. Such tracer development will provide novel imaging tools, if not theranostic leads, that should be beneficial not only for tumor imaging and cancer management but also for fundamental research investigating OTR’s role in health and disease.

While discoveries linking OTR to breast cancer are promising, only a single study evaluated OTR tracers in animals bearing *human* breast tumors, however, with poor pharmacological tracer characterization (no VPR data) and limited biodistribution info (no tumor/background tracer uptake ratios).^127^ Further *in vivo* studies using xenograft tumor models with different human breast cancer subtypes are needed to assess translational and clinical perspectives. OTR levels need to be systematically profiled across different subtypes to provide a clearer picture of OTR’s potential as a molecular target for imaging and therapy. OTR quantification at the protein level remains challenging due to the high extracellular homology of OTR with VPRs (V_1a_R, V_1b_R, and V_2_R) and a lack of specific antibodies.^157,159,184,185^ OTR tracers may be able to help in this task, given sufficient signal/brightness and selectivity and affinity to reliably quantify OTR in cells and tissue.

Peptide-based fluorescent tracers and radiotracers have certain opportunities and advantages over antibodies. Indeed, attempts to develop small molecules and biologics targeting OTR for clinical use have so far failed.^158^ First, peptides can be rapidly designed and chemically produced at reduced costs compared to antibodies.^186^ Peptides have good biocompatibility and low immunogenicity and can be modified to enhance the *in vivo* stability and pharmacokinetics. For instance, blood circulation of peptides can be increased through conjugation to polyethylene glycol (PEG) chains or serum albumin binders,^187^ which might enhance tumor accumulation. Since peptides are smaller than antibodies (∼3 kDa peptides vs ∼150 kDa antibodies), they have better tumor penetration; additionally, they can induce ligand–receptor-mediated internalization, which could lead to enhanced tumor uptake and visualization.^188^ This may be particularly important for targeted radiotherapy, where therapeutic radionuclides, e.g., ^90^Y or ^177^Lu, or chemotherapeutic agents would accumulate closer to the cancer cell nuclei than without internalization.^73,189,190^ Such targeted treatment is desirable for cancer subtypes that do not yet have a targeted therapy option (e.g., TNBC) and where systemic chemotherapy with its severe side effects remains the first-line treatment.^191^

This so-called peptide receptor radionucleotide therapy (PRRT) has already been successfully applied to neuroendocrine tumors (NETs) targeting the peptide hormone somatostatin receptor (SSTR).^192−194^ [^177^Lu]Lu-DOTA-TATE (Lutathera) was approved by the EMA in 2017 and the FDA in 2018 for treating SSTR-positive gastroenteropancreatic NETs.^195^ The success of somatostatin radiotracers paved the way for other peptide-based radiotracers, e.g., those based on the RGD peptide, vasoactive intestinal peptide (VIP), cholecystokinin (CCK)/gastrin peptide, α-melanocyte-stimulating hormone (α-MSH), neurotensin (NT), T140, exendin-4, neuropeptide Y (NPY), substance P, and tumor molecular targeted peptide 1 (TMTP1).^65,196,197^ For breast cancer management, chemerin-based [^68^Ga]Ga-DOTA peptide conjugates targeting the chemokine-like receptor 1 (CMKLR1) with high specificity and affinity could visualize CMKLR1-positive breast cancer xenografts via PET/MRI.^198^ [^68^Ga]Ga-NeoBOMB1, a DOTA-coupled GRPR antagonist, was tested in a study in four patients diagnosed with prostate cancer, where it rapidly localized in pathologic lesions, achieving high-contrast imaging during PET/CT.^199^ Breast cancer patients with GRPR-positive tumors are potential candidates for treatment with ^177^Lu-labeled NeoBOMB1.^200^ Taken together, these clinical trials support the safety and efficacy of peptide-based radiotracers and the theranostic opportunities of PRRT, providing a strong foundation for a thriving biotech industry that is already embracing these approaches and seeing the therapeutic potential of peptide-based theranostics.^201,202^

## 7 Conclusions and Perspectives

The landscape of breast cancer management has evolved significantly with the advent of targeted therapies, representing a marked departure from the era of indiscriminate chemotherapy or radiation treatments. This transformation translates into tangible benefits for patient survival, overall care, and the emergence of personalized treatment strategies. To further elevate these advancements, the focus must shift toward achieving earlier and more precise diagnoses, leveraging predictive biomarkers, and refining tumor-targeted therapies to mitigate side effects. Overcoming diagnostic challenges posed by the high heterogeneity of breast tumors and dense breast tissue requires an integrated approach, combining mammography with highly sensitive nuclear imaging techniques. Among these, PET stands out as a particularly promising modality, especially when it is coupled with cancer-specific radiotracers.

A notable stride in targeted therapies is the growing recognition of PRRT in the pharmaceutical realm, offering treatments that are both less toxic and more efficient, with recent approvals marking a pivotal milestone. PRRT underscores the advantages of employing peptide radiotracers in cancer treatment, characterized by their high target specificity, excellent tumor penetration, and swift clearance. Within this evolving landscape, OTR emerges as a novel target for breast cancer diagnosis and targeted therapy, particularly relevant in the context of TNBC. Despite its promise, more systematic studies are imperative to validate OTR in human breast cancer xenograft models and assess OTR tumor expression across diverse patient populations.

The development of OTR-specific ligands and tracers plays a critical role in advancing these studies, serving as molecular tools for investigating OTR in breast cancer and beyond. Recent advancements in OTR structural elucidation through X-ray and cryo-EM will enhance the rational designs of OTR-specific radiotracers, and previous studies highlight position 8 of OT as the optimal site for attaching tracer-related tags. It is important to underscore that, akin to successful PRRT approaches targeting overexpressed receptors in cancers (e.g., SSTR, GRPR), the efficacy and feasibility of OTR-targeted radiotherapy hinge on significant OTR overexpression in tumors and the OTR-selectivity of the radiotracers. Such OTR-selective radiotracers are poised to contribute substantially to the expanding frontier of enhancing cancer care—ranging from improved disease diagnosis and staging to precision therapy monitoring and tumor-targeted interventions with fewer side effects.

## Abbreviations Used

α-MSH: α-melanocyte-stimulating hormone
CCK: cholecystokinin
CMKLR1: chemokine-like receptor 1
EDDA: ethylenediaminediacetic acid
ER: estrogen receptor
GPCR: G protein-coupled receptor
GRPR: gastrin-releasing peptide receptor
HER2: human epidermal growth factor receptor 2
ICG: indocyanine green
MRI: magnetic resonance imaging
NET: neuroendocrine tumor
NIR: near-infrared
NPY: neuropeptide Y
NT: neurotensin
OT: oxytocin
OTR: oxytocin receptor
PD: pharmacodynamic
PD1: programmed cell death protein 1
PEG: polyethylene glycol
PET: positron emission tomography
PK: pharmacokinetic
PR: progesterone receptor
PRRT: peptide receptor radionucleotide therapy
SLNB: sentinel lymph node biopsy
SPECT: single-photon emission computed tomography
SSTR: somatostatin receptor
T-DM1: trastuzumab and ado-trastuzumab emtansine
T-Dxd: fam-trastuzumab deruxtecan-nxki
TM: transmembrane
TMTP1: tumor molecular targeted peptide 1
TNBC: triple-negative breast cancer
TYKERB: tyrosine kinase inhibitor lapatinib
VIP: vasoactive intestinal peptide
VIP-R1: vasoactive intestinal polypeptide receptor 1
VPR: vasopressin receptor

## References

[ref1] Bray F.; Ferlay J.; Soerjomataram I.; Siegel R. L.; Torre L. A.; Jemal A. Global Cancer Statistics 2018: GLOBOCAN Estimates of Incidence and Mortality Worldwide for 36 Cancers in 185 Countries. CA. Cancer J. Clin. 2018, 68 (6), 394–424. 10.3322/caac.21492. 30207593

[ref2] Sung H.; Ferlay J.; Siegel R. L.; Laversanne M.; Soerjomataram I.; Jemal A.; Bray F. Global Cancer Statistics 2020: GLOBOCAN Estimates of Incidence and Mortality Worldwide for 36 Cancers in 185 Countries. CA. Cancer J. Clin. 2021, 71 (3), 209–249. 10.3322/caac.21660. 33538338

[ref3] Arnold M.; Morgan E.; Rumgay H.; Mafra A.; Singh D.; Laversanne M.; Vignat J.; Gralow J. R.; Cardoso F.; Siesling S.; Soerjomataram I. Current and Future Burden of Breast Cancer: Global Statistics for 2020 and 2040. Breast Edinb. Scotl. 2022, 66, 15–23. 10.1016/j.breast.2022.08.010. PMC9465273 36084384

[ref4] Allemani C.; Matsuda T.; Di Carlo V.; Harewood R.; Matz M.; Nikšić M.; Bonaventure A.; Valkov M.; Johnson C. J.; Estève J.; Ogunbiyi O. J.; Azevedo e Silva G.; Chen W.-Q.; Eser S.; Engholm G.; Stiller C. A.; Monnereau A.; Woods R. R.; Visser O.; Lim G. H.; Aitken J.; Weir H. K.; Coleman M. P.; Bouzbid S.; Hamdi-Chérif M.; Zaidi Z.; Meguenni K.; Regagba D.; Bayo S.; Cheick Bougadari T.; Manraj S. S.; Bendahhou K.; Fabowale A.; Bradshaw D.; Somdyala N. I. M.; Kumcher I.; Moreno F.; Calabrano G. H.; Espinola S. B.; Carballo Quintero B.; Fita R.; Diumenjo M. C.; Laspada W. D.; Ibañez S. G.; Lima C. A.; De Souza P. C. F.; Del Pino K.; Laporte C.; Curado M. P.; de Oliveira J. C.; Veneziano C. L. A.; Veneziano D. B.; Latorre M. R. D. O.; Tanaka L. F.; Rebelo M. S.; Santos M. O.; Galaz J. C.; Aparicio Aravena M.; Sanhueza Monsalve J.; Herrmann D. A.; Vargas S.; Herrera V. M.; Uribe C. J.; Bravo L. E.; Garcia L. S.; Arias-Ortiz N. E.; Morantes D.; Jurado D. M.; Yépez Chamorro M. C.; Delgado S.; Ramirez M.; Galán Alvarez Y. H.; Torres P.; Martínez-Reyes F.; Jaramillo L.; Quinto R.; Castillo J.; Mendoza M.; Cueva P.; Yépez J. G.; Bhakkan B.; Deloumeaux J.; Joachim C.; Macni J.; Carrillo R.; Shalkow Klincovstein J.; Rivera Gomez R.; Poquioma E.; Tortolero-Luna G.; Zavala D.; Alonso R.; Barrios E.; Eckstrand A.; Nikiforuk C.; Noonan G.; Turner D.; Kumar E.; Zhang B.; McCrate F. R.; Ryan S.; MacIntyre M.; Saint-Jacques N.; Nishri D. E.; McClure C. A.; Vriends K. A.; Kozie S.; Stuart-Panko H.; Freeman T.; George J. T.; Brockhouse J. T.; O’Brien D. K.; Holt A.; Almon L.; Kwong S.; Morris C.; Rycroft R.; Mueller L.; Phillips C. E.; Brown H.; Cromartie B.; Schwartz A. G.; Vigneau F.; Levin G. M.; Wohler B.; Bayakly R.; Ward K. C.; Gomez S. L.; McKinley M.; Cress R.; Green M. D.; Miyagi K.; Ruppert L. P.; Lynch C. F.; Huang B.; Tucker T. C.; Deapen D.; Liu L.; Hsieh M. C.; Wu X. C.; Schwenn M.; Gershman S. T.; Knowlton R. C.; Alverson G.; Copeland G. E.; Bushhouse S.; Rogers D. B.; Jackson-Thompson J.; Lemons D.; Zimmerman H. J.; Hood M.; Roberts-Johnson J.; Rees J. R.; Riddle B.; Pawlish K. S.; Stroup A.; Key C.; Wiggins C.; Kahn A. R.; Schymura M. J.; Radhakrishnan S.; Rao C.; Giljahn L. K.; Slocumb R. M.; Espinoza R. E.; Khan F.; Aird K. G.; Beran T.; Rubertone J. J.; Slack S. J.; Garcia L.; Rousseau D. L.; Janes T. A.; Schwartz S. M.; Bolick S. W.; Hurley D. M.; Whiteside M. A.; Miller-Gianturco P.; Williams M. A.; Herget K.; Sweeney C.; Johnson A. T.; Keitheri Cheteri M. B.; Migliore Santiago P.; Blankenship S. E.; Farley S.; Borchers R.; Malicki R.; Espinoza J. R.; Grandpre J.; Wilson R.; Edwards B. K.; Mariotto A.; Lei Y.; Wang N.; Chen J. S.; Zhou Y.; He Y. T.; Song G. H.; Gu X. P.; Mei D.; Mu H. J.; Ge H. M.; Wu T. H.; Li Y. Y.; Zhao D. L.; Jin F.; Zhang J. H.; Zhu F. D.; Junhua Q.; Yang Y. L.; Jiang C. X.; Biao W.; Wang J.; Li Q. L.; Yi H.; Zhou X.; Dong J.; Li W.; Fu F. X.; Liu S. Z.; Chen J. G.; Zhu J.; Li Y. H.; Lu Y. Q.; Fan M.; Huang S. Q.; Guo G. P.; Zhaolai H.; Wei K.; Zeng H.; Demetriou A. V.; Mang W. K.; Ngan K. C.; Kataki A. C.; Krishnatreya M.; Jayalekshmi P. A.; Sebastian P.; Nandakumar A.; Malekzadeh R.; Roshandel G.; Keinan-Boker L.; Silverman B. G.; Ito H.; Nakagawa H.; Sato M.; Tobori F.; Nakata I.; Teramoto N.; Hattori M.; Kaizaki Y.; Moki F.; Sugiyama H.; Utada M.; Nishimura M.; Yoshida K.; Kurosawa K.; Nemoto Y.; Narimatsu H.; Sakaguchi M.; Kanemura S.; Naito M.; Narisawa R.; Miyashiro I.; Nakata K.; Sato S.; Yoshii M.; Oki I.; Fukushima N.; Shibata A.; Iwasa K.; Ono C.; Nimri O.; Jung K. W.; Won Y. J.; Alawadhi E.; Elbasmi A.; Ab Manan A.; Adam F.; Sanjaajmats E.; Tudev U.; Ochir C.; Al Khater A. M.; El Mistiri M. M.; Teo Y. Y.; Chiang C. J.; Lee W. C.; Buasom R.; Sangrajrang S.; Kamsa-ard S.; Wiangnon S.; Daoprasert K.; Pongnikorn D.; Leklob A.; Sangkitipaiboon S.; Geater S. L.; Sriplung H.; Ceylan O.; Kög I.; Dirican O.; Köse T.; Gurbuz T.; Karaşahin F. E.; Turhan D.; Aktaş U.; Halat Y.; Yakut C. I.; Altinisik M.; Cavusoglu Y.; Türkköylü A.; Üçüncü N.; Hackl M.; Zborovskaya A. A.; Aleinikova O. V.; Henau K.; Van Eycken L.; Valerianova Z.; Yordanova M. R.; Šekerija M.; Dušek L.; Zvolský M.; Storm H.; Innos K.; Mägi M.; Malila N.; Seppä K.; Jégu J.; Velten M.; Cornet E.; Troussard X.; Bouvier A. M.; Guizard A. V.; Bouvier V.; Launoy G.; Arveux P.; Maynadié M.; Mounier M.; Woronoff A. S.; Daoulas M.; Robaszkiewicz M.; Clavel J.; Goujon S.; Lacour B.; Baldi I.; Pouchieu C.; Amadeo B.; Coureau G.; Orazio S.; Preux P. M.; Rharbaoui F.; Marrer E.; Trétarre B.; Colonna M.; Delafosse P.; Ligier K.; Plouvier S.; Cowppli-Bony A.; Molinié F.; Bara S.; Ganry O.; Lapôtre-Ledoux B.; Grosclaude P.; Bossard N.; Uhry Z.; Bray F.; Piñeros M.; Stabenow R.; Wilsdorf-Köhler H.; Eberle A.; Luttmann S.; Löhden I.; Nennecke A. L.; Kieschke J.; Sirri E.; Emrich K.; Zeissig S. R.; Holleczek B.; Eisemann N.; Katalinic A.; Asquez R. A.; Kumar V.; Petridou E.; Ólafsdóttir E. J.; Tryggvadóttir L.; Clough-Gorr K.; Walsh P. M.; Sundseth H.; Mazzoleni G.; Vittadello F.; Coviello E.; Cuccaro F.; Galasso R.; Sampietro G.; Giacomin A.; Magoni M.; Ardizzone A.; D’Argenzio A.; Castaing M.; Grosso G.; Lavecchia A. M.; Sutera Sardo A.; Gola G.; Gatti L.; Ricci P.; Ferretti S.; Serraino D.; Zucchetto A.; Celesia M. V.; Filiberti R. A.; Pannozzo F.; Melcarne A.; Quarta F.; Russo A. G.; Carrozzi G.; Cirilli C.; Cavalieri d’Oro L.; Rognoni M.; Fusco M.; Vitale M. F.; Usala M.; Cusimano R.; Mazzucco W.; Michiara M.; Sgargi P.; Boschetti L.; Borciani E.; Seghini P.; Maule M. M.; Merletti F.; Tumino R.; Mancuso P.; Vicentini M.; Cassetti T.; Sassatelli R.; Falcini F.; Giorgetti S.; Caiazzo A. L.; Cavallo R.; Cesaraccio R.; Pirino D. R.; Contrino M. L.; Tisano F.; Fanetti A. C.; Maspero S.; Carone S.; Mincuzzi A.; Candela G.; Scuderi T.; Gentilini M. A.; Piffer S.; Rosso S.; Barchielli A.; Caldarella A.; Bianconi F.; Stracci F.; Contiero P.; Tagliabue G.; Rugge M.; Zorzi M.; Beggiato S.; Brustolin A.; Berrino F.; Gatta G.; Sant M.; Buzzoni C.; Mangone L.; Capocaccia R.; De Angelis R.; Zanetti R.; Maurina A.; Pildava S.; Lipunova N.; Vincerževskiené I.; Agius D.; Calleja N.; Siesling S.; Larønningen S.; Møller B.; Dyzmann-Sroka A.; Trojanowski M.; Góźdoź S.; Mężyk R.; Mierzwa T.; Molong L.; Rachtan J.; Szewczyk S.; Błaszczyk J.; Kępska K.; Kościańska B.; Tarocińska K.; Zwierko M.; Drosik K.; Maksimowicz K. M.; Purwin-Porowska E.; Reca E.; Wójcik-Tomaszewska J.; Tukiendorf A.; Grądalska-Lampart M.; Radziszewska A. U.; Gos A.; Talerczyk M.; Wyborska M.; Didkowska J. A.; Wojciechowska U.; Bielska-Lasota M.; Forjaz de Lacerda G.; Rego R. A.; Bastos J.; Silva M. A.; Antunes L.; Laranja Pontes J.; Mayer-da-Silva A.; Miranda A.; Blaga L. M.; Coza D.; Gusenkova L.; Lazarevich O.; Prudnikova O.; Vjushkov D. M.; Egorova A. G.; Orlov A. E.; Kudyakov L. A.; Pikalova L. V.; Adamcik J.; Safaei Diba C.; Primic-Žakelj M.; Zadnik V.; Larrañaga N.; Lopez de Munain A.; Herrera A. A.; Redondas R.; Marcos-Gragera R.; Vilardell Gil M. L.; Molina E.; Sánchez Perez M. J.; Franch Sureda P.; Ramos Montserrat M.; Chirlaque M. D.; Navarro C.; Ardanaz E. E.; Guevara M. M.; Fernández-Delgado R.; Peris-Bonet R.; Carulla M.; Galceran J.; Alberich C.; Vicente-Raneda M.; Khan S.; Pettersson D.; Dickman P.; Avelina I.; Staehelin K.; Camey B.; Bouchardy C.; Schaffar R.; Frick H.; Herrmann C.; Bulliard J. L.; Maspoli-Conconi M.; Kuehni C. E.; Redmond S. M.; Bordoni A.; Ortelli L.; Chiolero A.; Konzelmann I.; Matthes K. L.; Rohrmann S.; Broggio J.; Rashbass J.; Fitzpatrick D.; Gavin A.; Clark D. I.; Deas A. J.; Huws D. W.; White C.; Montel L.; Rachet B.; Turculet A. D.; Stephens R.; Chalker E.; Phung H.; Walton R.; You H.; Guthridge S.; Johnson F.; Gordon P.; D’Onise K.; Priest K.; Stokes B. C.; Venn A.; Farrugia H.; Thursfield V.; Dowling J.; Currow D.; Hendrix J.; Lewis C. Global Surveillance of Trends in Cancer Survival 2000–14 (CONCORD-3): Analysis of Individual Records for 37 513 025 Patients Diagnosed with One of 18 Cancers from 322 Population-Based Registries in 71 Countries. Lancet 2018, 391 (10125), 1023–1075. 10.1016/S0140-6736(17)33326-3. 29395269 PMC5879496

[ref5] Turashvili G.; Brogi E. Tumor Heterogeneity in Breast Cancer. Front. Med. 2017, 4, 227 10.3389/fmed.2017.00227. PMC5727049 29276709

[ref6] Bianchini G.; Balko J. M.; Mayer I. A.; Sanders M. E.; Gianni L. Triple-Negative Breast Cancer: Challenges and Opportunities of a Heterogeneous Disease. Nat. Rev. Clin. Oncol. 2016, 13 (11), 674–690. 10.1038/nrclinonc.2016.66. 27184417 PMC5461122

[ref7] Paik S.; Tang G.; Shak S.; Kim C.; Baker J.; Kim W.; Cronin M.; Baehner F. L.; Watson D.; Bryant J.; Costantino J. P.; Geyer C. E.; Wickerham D. L.; Wolmark N. Gene Expression and Benefit of Chemotherapy in Women With Node-Negative, Estrogen Receptor-Positive Breast Cancer. J. Clin. Oncol. 2006, 24 (23), 3726–3734. 10.1200/JCO.2005.04.7985. 16720680

[ref8] Nishimura R.; Osako T.; Okumura Y.; Hayashi M.; Toyozumi Y.; Arima N. Ki-67 as a Prognostic Marker According to Breast Cancer Subtype and a Predictor of Recurrence Time in Primary Breast Cancer. Exp. Ther. Med. 2010, 1 (5), 747–754. 10.3892/etm.2010.133. 22993598 PMC3445951

[ref9] Orrantia-Borunda E.; Anchondo-Nuñez P.; Acuña-Aguilar L. E.; Gómez-Valles F. O.; Ramírez-Valdespino C. A. Subtypes of Breast Cancer. In Breast Cancer; Mayrovitz H. N., Ed.; Exon Publications: Brisbane, 2022. 36122153

[ref10] Merino Bonilla J. A.; Torres Tabanera M.; Ros Mendoza L. H. Breast Cancer in the 21st Century: From Early Detection to New Therapies. Radiol. Engl. Ed. 2017, 59 (5), 368–379. 10.1016/j.rxeng.2017.08.001. 28712528

[ref11] Sauter E. R. Reliable Biomarkers to Identify New and Recurrent Cancer. Eur. J. Breast Health 2017, 13 (4), 162–167. 10.5152/ejbh.2017.3635. 29082372 PMC5648271

[ref12] Schlam I.; Swain S. M. HER2-Positive Breast Cancer and Tyrosine Kinase Inhibitors: The Time Is Now. NPJ. Breast Cancer 2021, 7 (1), 56 10.1038/s41523-021-00265-1. 34016991 PMC8137941

[ref13] Najjar M. K.; Manore S. G.; Regua A. T.; Lo H.-W. Antibody-Drug Conjugates for the Treatment of HER2-Positive Breast Cancer. Genes 2022, 13 (11), 2065 10.3390/genes13112065. 36360302 PMC9691220

[ref14] Toft D. J.; Cryns V. L. Minireview: Basal-Like Breast Cancer: From Molecular Profiles to Targeted Therapies. Mol. Endocrinol. 2011, 25 (2), 199–211. 10.1210/me.2010-0164. 20861225 PMC3035993

[ref15] Lehmann B. D.; Pietenpol J. A.; Tan A. R. Triple-Negative Breast Cancer: Molecular Subtypes and New Targets for Therapy. Am. Soc. Clin. Oncol. Educ. Book 2015, (35), e31–e39. 10.14694/EdBook_AM.2015.35.e31. 25993190

[ref16] Podo F.; Buydens L. M. C.; Degani H.; Hilhorst R.; Klipp E.; Gribbestad I. S.; Van Huffel S.; van Laarhoven H. W. M.; Luts J.; Monleon D.; Postma G. J.; Schneiderhan-Marra N.; Santoro F.; Wouters H.; Russnes H. G.; Sørlie T.; Tagliabue E.; Børresen-Dale A.-L. Triple-Negative Breast Cancer: Present Challenges and New Perspectives. Mol. Oncol. 2010, 4 (3), 209–229. 10.1016/j.molonc.2010.04.006. 20537966 PMC5527939

[ref17] Foulkes W. D.; Smith I. E.; Reis-Filho J. S. Triple-Negative Breast Cancer. N. Engl. J. Med. 2010, 363 (20), 1938–1948. 10.1056/NEJMra1001389. 21067385

[ref18] Nielsen T. O.; Hsu F. D.; Jensen K.; Cheang M.; Karaca G.; Hu Z.; Hernandez-Boussard T.; Livasy C.; Cowan D.; Dressler L.; Akslen L. A.; Ragaz J.; Gown A. M.; Gilks C. B.; van de Rijn M.; Perou C. M. Immunohistochemical and Clinical Characterization of the Basal-Like Subtype of Invasive Breast Carcinoma. Clin. Cancer Res. 2004, 10 (16), 5367–5374. 10.1158/1078-0432.CCR-04-0220. 15328174

[ref19] Shah M.; Osgood C. L.; Amatya A. K.; Fiero M. H.; Pierce W. F.; Nair A.; Herz J.; Robertson K. J.; Mixter B. D.; Tang S.; Pazdur R.; Beaver J. A.; Amiri-Kordestani L. FDA Approval Summary: Pembrolizumab for Neoadjuvant and Adjuvant Treatment of Patients with High-Risk Early-Stage Triple-Negative Breast Cancer. Clin. Cancer Res. 2022, 28 (24), 5249–5253. 10.1158/1078-0432.CCR-22-1110. 35925043

[ref20] Kwok G.; Yau T. C. C.; Chiu J. W.; Tse E.; Kwong Y.-L. Pembrolizumab (Keytruda). Hum. Vaccines Immunother. 2016, 12 (11), 2777–2789. 10.1080/21645515.2016.1199310. PMC5137544 27398650

[ref21] Liu H.; Muttenthaler M. High Oxytocin Receptor Expression Linked to Increased Cell Migration and Reduced Survival in Patients with Triple-Negative Breast Cancer. Biomedicines 2022, 10 (7), 1595 10.3390/biomedicines10071595. 35884900 PMC9313263

[ref22] Weigelt B.; Baehner F. L.; Reis-Filho J. S. The Contribution of Gene Expression Profiling to Breast Cancer Classification, Prognostication and Prediction: A Retrospective of the Last Decade: A Commentary on Microarrays in Breast Cancer Research. J. Pathol. 2010, 220 (2), 263–280. 10.1002/path.2648. 19927298

[ref23] Ades F.; Zardavas D.; Bozovic-Spasojevic I.; Pugliano L.; Fumagalli D.; de Azambuja E.; Viale G.; Sotiriou C.; Piccart M. Luminal B Breast Cancer: Molecular Characterization, Clinical Management, and Future Perspectives. J. Clin. Oncol. 2014, 32 (25), 2794–2803. 10.1200/JCO.2013.54.1870. 25049332

[ref24] Masuda H.; Baggerly K. A.; Wang Y.; Zhang Y.; Gonzalez-Angulo A. M.; Meric-Bernstam F.; Valero V.; Lehmann B. D.; Pietenpol J. A.; Hortobagyi G. N.; Symmans W. F.; Ueno N. T. Differential Response to Neoadjuvant Chemotherapy Among 7 Triple-Negative Breast Cancer Molecular Subtypes. Clin. Cancer Res. 2013, 19 (19), 5533–5540. 10.1158/1078-0432.CCR-13-0799. 23948975 PMC3813597

[ref25] Provencher L.; Hogue J. C.; Desbiens C.; Poirier B.; Poirier E.; Boudreau D.; Joyal M.; Diorio C.; Duchesne N.; Chiquette J. Is Clinical Breast Examination Important for Breast Cancer Detection?. Curr. Oncol. 2016, 23 (4), 332–339. 10.3747/co.23.2881. PMC4974039 27536182

[ref26] Gøtzsche P. C.; Jørgensen K. J. Screening for Breast Cancer with Mammography. Cochrane Database Syst. Rev. 2013, 2013 (6), CD001877 10.1002/14651858.CD001877.pub5. 23737396 PMC6464778

[ref27] Kerlikowske K. Efficacy of Screening Mammography Among Women Aged 40 to 49 Years and 50 to 69 Years: Comparison of Relative and Absolute Benefit. JNCI Monogr. 1997, 1997 (22), 79–86. 10.1093/jncimono/1997.22.79. 9709281

[ref28] Buist D. S. M.; Porter P. L.; Lehman C.; Taplin S. H.; White E. Factors Contributing to Mammography Failure in Women Aged 40–49 Years. JNCI J. Natl. Cancer Inst. 2004, 96 (19), 1432–1440. 10.1093/jnci/djh269. 15467032

[ref29] Yerushalmy J. Statistical Problems in Assessing Methods of Medical Diagnosis, with Special Reference to X-Ray Techniques. Public Health Rep. 1896–1970 1947, 62 (40), 1432 10.2307/4586294. 20340527

[ref30] Pinsky R. W.; Helvie M. A. Mammographic Breast Density: Effect on Imaging and Breast Cancer Risk. J. Natl. Compr. Canc. Netw. 2010, 8 (10), 1157–1165. 10.6004/jnccn.2010.0085. 20971840

[ref31] Kolb T. M.; Lichy J.; Newhouse J. H. Comparison of the Performance of Screening Mammography, Physical Examination, and Breast US and Evaluation of Factors That Influence Them: An Analysis of 27,825 Patient Evaluations. Radiology 2002, 225 (1), 165–175. 10.1148/radiol.2251011667. 12355001

[ref32] Morrow M.; Waters J.; Morris E. MRI for Breast Cancer Screening, Diagnosis, and Treatment. Lancet 2011, 378 (9805), 1804–1811. 10.1016/S0140-6736(11)61350-0. 22098853

[ref33] Hooley R. J.; Scoutt L. M.; Philpotts L. E. Breast Ultrasonography: State of the Art. Radiology 2013, 268 (3), 642–659. 10.1148/radiol.13121606. 23970509

[ref34] Taskin F.; Polat Y.; Erdogdu I. H.; Turkdogan F. T.; Ozturk V. S.; Ozbas S. Problem-Solving Breast MRI: Useful or a Source of New Problems?. Diagn. Interv. Radiol. 2018, 24 (5), 255–261. 10.5152/dir.2018.17504. 30211678 PMC6135053

[ref35] Krag D. N.; Julian T. B.; Harlow S. P.; Weaver D. L.; Ashikaga T.; Bryant J.; Single R. M.; Wolmark N. NSABP-32: Phase III, Randomized Trial Comparing Axillary Resection with Sentinal Lymph Node Dissection: A Description of the Trial. Ann. Surg. Oncol. 2004, 11 (S3), 208S–210S. 10.1007/BF02523630. 15023753

[ref36] Tang G.; Shak S.; Paik S.; Anderson S. J.; Costantino J. P.; Geyer C. E.; Mamounas E. P.; Wickerham D. L.; Wolmark N. Comparison of the Prognostic and Predictive Utilities of the 21-Gene Recurrence Score Assay and Adjuvant! For Women with Node-Negative, ER-Positive Breast Cancer: Results from NSABP B-14 and NSABP B-20. Breast Cancer Res. Treat. 2011, 127 (1), 133–142. 10.1007/s10549-010-1331-z. 21221771 PMC4266581

[ref37] Dalm S.; Verzijlbergen J.; De Jong M. Review: Receptor Targeted Nuclear Imaging of Breast Cancer. Int. J. Mol. Sci. 2017, 18 (2), 260 10.3390/ijms18020260. 28134770 PMC5343796

[ref38] Barbosa A. M.; Martel F. Targeting Glucose Transporters for Breast Cancer Therapy: The Effect of Natural and Synthetic Compounds. Cancers 2020, 12 (1), 154 10.3390/cancers12010154. 31936350 PMC7016663

[ref39] Shin E.; Koo J. S. Glucose Metabolism and Glucose Transporters in Breast Cancer. Front. Cell Dev. Biol. 2021, 9, 728759 10.3389/fcell.2021.728759. 34552932 PMC8450384

[ref40] Cha Y.; Kim E.-S.; Koo J. Amino Acid Transporters and Glutamine Metabolism in Breast Cancer. Int. J. Mol. Sci. 2018, 19 (3), 907 10.3390/ijms19030907. 29562706 PMC5877768

[ref41] Zhang Y.; Wang J. Targeting Uptake Transporters for Cancer Imaging and Treatment. Acta Pharm. Sin. B 2020, 10 (1), 79–90. 10.1016/j.apsb.2019.12.005. 31993308 PMC6977162

[ref42] Crişan G.; Moldovean-Cioroianu N. S.; Timaru D.-G.; Andrieş G.; Căinap C.; Chiş V. Radiopharmaceuticals for PET and SPECT Imaging: A Literature Review over the Last Decade. Int. J. Mol. Sci. 2022, 23 (9), 5023 10.3390/ijms23095023. 35563414 PMC9103893

[ref43] Gøtzsche P. C.; Olsen O. Is Screening for Breast Cancer with Mammography Justifiable?. Lancet 2000, 355 (9198), 129–134. 10.1016/S0140-6736(99)06065-1. 10675181

[ref44] Gøtzsche P. C.; Nielsen M. Screening for Breast Cancer with Mammography. Cochrane Database Syst. Rev. 2011, 19, CD001877 10.1002/14651858.CD001877.pub4. 21249649

[ref45] Guo R.; Lu G.; Qin B.; Fei B. Ultrasound Imaging Technologies for Breast Cancer Detection and Management: A Review. Ultrasound Med. Biol. 2018, 44 (1), 37–70. 10.1016/j.ultrasmedbio.2017.09.012. 29107353 PMC6169997

[ref46] Shokuhi S.; Parker S. J. Management of Breast Cancer: Basic Principles. Surg. Oxf. 2007, 25 (6), 261–263. 10.1016/j.mpsur.2007.05.008.

[ref47] Akram M.; Iqbal M.; Daniyal M.; Khan A. U. Awareness and Current Knowledge of Breast Cancer. Biol. Res. 2017, 50 (1), 33 10.1186/s40659-017-0140-9. 28969709 PMC5625777

[ref48] Vallabhajosula S. Molecular Imaging: Radiopharmaceuticals for PET and SPECT; Springer-Verlag: Berlin, New York, 2009.

[ref49] Adak S.; Bhalla R.; Vijaya Raj K. K.; Mandal S.; Pickett R.; Luthra S. K. Radiotracers for SPECT Imaging: Current Scenario and Future Prospects. ract 2012, 100 (2), 95–107. 10.1524/ract.2011.1891.

[ref50] Almubarak M.; Osman S.; Marano G.; Abraham J. Role of Positron-Emission Tomography Scan in the Diagnosis and Management of Breast Cancer. Oncology (Williston Park) 2009, 23 (3), 255–261. 19418826

[ref51] Paik S.; Shak S.; Tang G.; Kim C.; Baker J.; Cronin M.; Baehner F. L.; Walker M. G.; Watson D.; Park T.; Hiller W.; Fisher E. R.; Wickerham D. L.; Bryant J.; Wolmark N. A Multigene Assay to Predict Recurrence of Tamoxifen-Treated, Node-Negative Breast Cancer. N. Engl. J. Med. 2004, 351 (27), 2817–2826. 10.1056/NEJMoa041588. 15591335

[ref52] Cardoso F.; van’t Veer L. J.; Bogaerts J.; Slaets L.; Viale G.; Delaloge S.; Pierga J.-Y.; Brain E.; Causeret S.; DeLorenzi M.; Glas A. M.; Golfinopoulos V.; Goulioti T.; Knox S.; Matos E.; Meulemans B.; Neijenhuis P. A.; Nitz U.; Passalacqua R.; Ravdin P.; Rubio I. T.; Saghatchian M.; Smilde T. J.; Sotiriou C.; Stork L.; Straehle C.; Thomas G.; Thompson A. M.; van der Hoeven J. M.; Vuylsteke P.; Bernards R.; Tryfonidis K.; Rutgers E.; Piccart M. 70-Gene Signature as an Aid to Treatment Decisions in Early-Stage Breast Cancer. N. Engl. J. Med. 2016, 375 (8), 717–729. 10.1056/NEJMoa1602253. 27557300

[ref53] Wallden B.; Storhoff J.; Nielsen T.; Dowidar N.; Schaper C.; Ferree S.; Liu S.; Leung S.; Geiss G.; Snider J.; Vickery T.; Davies S. R.; Mardis E. R.; Gnant M.; Sestak I.; Ellis M. J.; Perou C. M.; Bernard P. S.; Parker J. S. Development and Verification of the PAM50-Based Prosigna Breast Cancer Gene Signature Assay. BMC Med. Genomics 2015, 8 (1), 54 10.1186/s12920-015-0129-6. 26297356 PMC4546262

[ref54] Blok E. J.; Bastiaannet E.; van den Hout W. B.; Liefers G. J.; Smit V. T. H. B. M.; Kroep J. R.; van de Velde C. J. H. Systematic Review of the Clinical and Economic Value of Gene Expression Profiles for Invasive Early Breast Cancer Available in Europe. Cancer Treat. Rev. 2018, 62, 74–90. 10.1016/j.ctrv.2017.10.012. 29175678

[ref55] Hannouf M. B.; Zaric G. S.; Blanchette P.; Brezden-Masley C.; Paulden M.; McCabe C.; Raphael J.; Brackstone M. Cost-Effectiveness Analysis of Multigene Expression Profiling Assays to Guide Adjuvant Therapy Decisions in Women with Invasive Early-Stage Breast Cancer. Pharmacogenomics J. 2020, 20 (1), 27–46. 10.1038/s41397-019-0089-x. 31130722

[ref56] National Research Council (US) and Institute of Medicine (US) Committee on the Mathematics and Physics of Emerging Dynamic Biomedical Imaging Mathematics and Physics of Emerging Biomedical Imaging; National Academies Press: Washington, DC, 1996. 25121300

[ref57] Bénard F.; Turcotte É. Imaging in Breast Cancer: Single-Photon Computed Tomography and Positron-Emission Tomography. Breast Cancer Res. 2005, 7 (4), 153 10.1186/bcr1201. 15987467 PMC1175073

[ref58] Sergieva S.; Alexandrova E.; Baitchev G.; Parvanova V. SPECT-CT in Breast Cancer. Arch. Oncol. 2012, 20 (3–4), 127–131. 10.2298/AOO1204127S.

[ref59] Ziegler S. I. Positron Emission Tomography: Principles, Technology, and Recent Developments. Nucl. Phys. A 2005, 752, 679–687. 10.1016/j.nuclphysa.2005.02.067.

[ref60] Avril N.; Rosé C. A.; Schelling M.; Dose J.; Kuhn W.; Bense S.; Weber W.; Ziegler S.; Graeff H.; Schwaiger M. Breast Imaging With Positron Emission Tomography and Fluorine-18 Fluorodeoxyglucose: Use and Limitations. J. Clin. Oncol. 2000, 18 (20), 3495–3502. 10.1200/JCO.2000.18.20.3495. 11032590

[ref61] Yang S. K.; Cho N.; Moon W. K. The Role of PET/CT for Evaluating Breast Cancer. Korean J. Radiol. 2007, 8 (5), 429 10.3348/kjr.2007.8.5.429. 17923786 PMC2626817

[ref62] Erdi Y. E. Limits of Tumor Detectability in Nuclear Medicine and PET. Mol. Imaging Radionucl. Ther. 2012, 21 (2), 23–28. 10.4274/Mirt.138. 23486256 PMC3590963

[ref63] Narod S. A.; Iqbal J.; Jakubowska A.; Huzarski T.; Sun P.; Cybulski C.; Gronwald J.; Byrski T.; Lubinski J. Are Two-Centimeter Breast Cancers Large or Small?. Curr. Oncol. 2013, 20 (4), 205–211. 10.3747/co.20.1364. 23904761 PMC3728051

[ref64] Tafra L. Positron Emission Tomography (PET) and Mammography (PEM) for Breast Cancer: Importance to Surgeons. Ann. Surg. Oncol. 2006, 14 (1), 3–13. 10.1245/s10434-006-9019-7. 17066235

[ref65] Sun X.; Li Y.; Liu T.; Li Z.; Zhang X.; Chen X. Peptide-Based Imaging Agents for Cancer Detection. Adv. Drug Delivery Rev. 2017, 110–111, 38–51. 10.1016/j.addr.2016.06.007. PMC5235994 27327937

[ref66] Li J.; Van Valkenburgh J.; Hong X.; Conti P. S.; Zhang X.; Chen K. Small Molecules as Theranostic Agents in Cancer Immunology. Theranostics 2019, 9 (25), 7849–7871. 10.7150/thno.37218. 31695804 PMC6831453

[ref67] Di L. Strategic Approaches to Optimizing Peptide ADME Properties. AAPS J. 2015, 17 (1), 134–143. 10.1208/s12248-014-9687-3. 25366889 PMC4287298

[ref68] Schottelius M.; Wester H.-J. Molecular Imaging Targeting Peptide Receptors. Methods 2009, 48 (2), 161–177. 10.1016/j.ymeth.2009.03.012. 19324088

[ref69] Sachdeva S.; Joo H.; Tsai J.; Jasti B.; Li X. A Rational Approach for Creating Peptides Mimicking Antibody Binding. Sci. Rep. 2019, 9 (1), 997 10.1038/s41598-018-37201-6. 30700733 PMC6353898

[ref70] Muttenthaler M.; King G. F.; Adams D. J.; Alewood P. F. Trends in Peptide Drug Discovery. Nat. Rev. Drug Discovery 2021, 20 (4), 309–325. 10.1038/s41573-020-00135-8. 33536635

[ref71] Lee P.; Wu X. Review: Modifications of Human Serum Albumin and Their Binding Effect. Curr. Pharm. Des. 2015, 21 (14), 1862–1865. 10.2174/1381612821666150302115025. 25732553 PMC4654954

[ref72] van Leeuwen F. W. B.; Hardwick J. C. H.; van Erkel A. R. Luminescence-Based Imaging Approaches in the Field of Interventional Molecular Imaging. Radiology 2015, 276 (1), 12–29. 10.1148/radiol.2015132698. 26101919

[ref73] Mező G.; Manea M. Receptor-Mediated Tumor Targeting Based on Peptide Hormones. Expert Opin. Drug Delivery 2010, 7 (1), 79–96. 10.1517/17425240903418410. 19947889

[ref74] Johnsen S. Hidden in Plain Sight: The Ecology and Physiology of Organismal Transparency. Biol. Bull. 2001, 201 (3), 301–318. 10.2307/1543609. 11751243

[ref75] Hong G.; Antaris A. L.; Dai H. Near-Infrared Fluorophores for Biomedical Imaging. Nat. Biomed. Eng. 2017, 1 (1), 0010 10.1038/s41551-016-0010.

[ref76] Hussain T.; Nguyen Q. T. Molecular Imaging for Cancer Diagnosis and Surgery. Adv. Drug Delivery Rev. 2014, 66, 90–100. 10.1016/j.addr.2013.09.007. PMC4464660 24064465

[ref77] Huang J.; Pu K. Near-Infrared Fluorescent Molecular Probes for Imaging and Diagnosis of Nephro-Urological Diseases. Chem. Sci. 2021, 12 (10), 3379–3392. 10.1039/D0SC02925D. PMC8179423 34163613

[ref78] Agnes R. S.; Broome A.-M.; Wang J.; Verma A.; Lavik K.; Basilion J. P. An Optical Probe for Noninvasive Molecular Imaging of Orthotopic Brain Tumors Overexpressing Epidermal Growth Factor Receptor. Mol. Cancer Ther. 2012, 11 (10), 2202–2211. 10.1158/1535-7163.MCT-12-0211. 22807580 PMC3829608

[ref79] Cao J.; Wan S.; Tian J.; Li S.; Deng D.; Qian Z.; Gu Y. Fast Clearing RGD-Based near-Infrared Fluorescent Probes for in Vivo Tumor Diagnosis: Fast Clearing RGD-Based near-Infrared Probes. Contrast Media Mol. Imaging 2012, 7 (4), 390–402. 10.1002/cmmi.1464. 22649045

[ref80] Ding F.; Zhan Y.; Lu X.; Sun Y. Recent Advances in Near-Infrared II Fluorophores for Multifunctional Biomedical Imaging. Chem. Sci. 2018, 9 (19), 4370–4380. 10.1039/C8SC01153B. 29896378 PMC5961444

[ref81] Zhu S.; Hu Z.; Tian R.; Yung B. C.; Yang Q.; Zhao S.; Kiesewetter D. O.; Niu G.; Sun H.; Antaris A. L.; Chen X. Repurposing Cyanine NIR-I Dyes Accelerates Clinical Translation of Near-Infrared-II (NIR-II) Bioimaging. Adv. Mater. 2018, 30 (34), 1802546 10.1002/adma.201802546. 29985542

[ref82] Patil C. G.; Walker D. G.; Miller D. M.; Butte P.; Morrison B.; Kittle D. S.; Hansen S. J.; Nufer K. L.; Byrnes-Blake K. A.; Yamada M.; Lin L. L.; Pham K.; Perry J.; Parrish-Novak J.; Ishak L.; Prow T.; Black K.; Mamelak A. N. Phase 1 Safety, Pharmacokinetics, and Fluorescence Imaging Study of Tozuleristide (BLZ-100) in Adults With Newly Diagnosed or Recurrent Gliomas. Neurosurgery 2019, 85 (4), E641–E649. 10.1093/neuros/nyz125. 31069381

[ref83] Dintzis S. M.; Hansen S.; Harrington K. M.; Tan L. C.; Miller D. M.; Ishak L.; Parrish-Novak J.; Kittle D.; Perry J.; Gombotz C.; Fortney T.; Porenta S.; Hales L.; Calhoun K. E.; Anderson B. O.; Javid S. H.; Byrd D. R. Real-Time Visualization of Breast Carcinoma in Pathology Specimens From Patients Receiving Fluorescent Tumor-Marking Agent Tozuleristide. Arch. Pathol. Lab. Med. 2019, 143 (9), 1076–1083. 10.5858/arpa.2018-0197-OA. 30550350 PMC11781288

[ref84] Kalinyak J. E.; Berg W. A.; Schilling K.; Madsen K. S.; Narayanan D.; Tartar M. Breast Cancer Detection Using High-Resolution Breast PET Compared to Whole-Body PET or PET/CT. Eur. J. Nucl. Med. Mol. Imaging 2014, 41 (2), 260–275. 10.1007/s00259-013-2553-1. 24085500

[ref85] Hsu D. F. C.; Freese D. L.; Levin C. S. Breast-Dedicated Radionuclide Imaging Systems. J. Nucl. Med. 2016, 57 (Supplement 1), 40S–45S. 10.2967/jnumed.115.157883. 26834101

[ref86] Mintun M. A.; Welch M. J.; Siegel B. A.; Mathias C. J.; Brodack J. W.; McGuire A. H.; Katzenellenbogen J. A. Breast Cancer: PET Imaging of Estrogen Receptors. Radiology 1988, 169 (1), 45–48. 10.1148/radiology.169.1.3262228. 3262228

[ref87] Peterson L. M.; Mankoff D. A.; Lawton T.; Yagle K.; Schubert E. K.; Stekhova S.; Gown A.; Link J. M.; Tewson T.; Krohn K. A. Quantitative Imaging of Estrogen Receptor Expression in Breast Cancer with PET and ^18^ F-Fluoroestradiol. J. Nucl. Med. 2008, 49 (3), 367–374. 10.2967/jnumed.107.047506. 18287268

[ref88] Mortimer J. E.; Dehdashti F.; Siegel B. A.; Katzenellenbogen J. A.; Fracasso P.; Welch M. J. Positron Emission Tomography with 2-[18F]Fluoro-2-Deoxy-D-Glucose and 16alpha-[18F]Fluoro-17beta-Estradiol in Breast Cancer: Correlation with Estrogen Receptor Status and Response to Systemic Therapy. Clin. Cancer Res. 1996, 2 (6), 933–939. 9816253

[ref89] Dehdashti F.; Mortimer J. E.; Siegel B. A.; Griffeth L. K.; Bonasera T. J.; Fusselman M. J.; Detert D. D.; Cutler P. D.; Katzenellenbogen J. A.; Welch M. J. Positron Tomographic Assessment of Estrogen Receptors in Breast Cancer: Comparison with FDG-PET and in Vitro Receptor Assays. J. Nucl. Med. 1995, 36 (10), 1766–1774. 7562040

[ref90] Gemignani M. L.; Patil S.; Seshan V. E.; Sampson M.; Humm J. L.; Lewis J. S.; Brogi E.; Larson S. M.; Morrow M.; Pandit-Taskar N. Feasibility and Predictability of Perioperative PET and Estrogen Receptor Ligand in Patients with Invasive Breast Cancer. J. Nucl. Med. 2013, 54 (10), 1697–1702. 10.2967/jnumed.112.113373. 23970364 PMC4404505

[ref91] Kiesewetter D. O.; Kilbourn M. R.; Landvatter S. W.; Heiman D. F.; Katzenellenbogen J. A.; Welch M. J. Preparation of Four Fluorine-18-Labeled Estrogens and Their Selective Uptakes in Target Tissues of Immature Rats. J. Nucl. Med. 1984, 25 (11), 1212–1221. 6092569

[ref92] He S.; Wang M.; Yang Z.; Zhang J.; Zhang Y.; Luo J.; Zhang Y. Comparison of 18F-FES, 18F-FDG, and 18F-FMISO PET Imaging Probes for Early Prediction and Monitoring of Response to Endocrine Therapy in a Mouse Xenograft Model of ER-Positive Breast Cancer. PLoS One 2016, 11 (7), e0159916 10.1371/journal.pone.0159916. 27467716 PMC4965120

[ref93] Dehdashti F.; Laforest R.; Gao F.; Aft R. L.; Dence C. S.; Zhou D.; Shoghi K. I.; Siegel B. A.; Katzenellenbogen J. A.; Welch M. J. Assessment of Progesterone Receptors in Breast Carcinoma by PET with 21- ^18^ F-Fluoro-16α,17α-[(*R*)-(1′-α-Furylmethylidene)Dioxy]-19-Norpregn-4-Ene-3,20-Dione. J. Nucl. Med. 2012, 53 (3), 363–370. 10.2967/jnumed.111.098319. 22331216 PMC3595048

[ref94] Carey L. A.; Perou C. M.; Livasy C. A.; Dressler L. G.; Cowan D.; Conway K.; Karaca G.; Troester M. A.; Tse C. K.; Edmiston S.; Deming S. L.; Geradts J.; Cheang M. C. U.; Nielsen T. O.; Moorman P. G.; Earp H. S.; Millikan R. C. Race, Breast Cancer Subtypes, and Survival in the Carolina Breast Cancer Study. JAMA 2006, 295 (21), 2492 10.1001/jama.295.21.2492. 16757721

[ref95] Kurihara H.; Hamada A.; Yoshida M.; Shimma S.; Hashimoto J.; Yonemori K.; Tani H.; Miyakita Y.; Kanayama Y.; Wada Y.; Kodaira M.; Yunokawa M.; Yamamoto H.; Shimizu C.; Takahashi K.; Watanabe Y.; Fujiwara Y.; Tamura K. 64Cu-DOTA-Trastuzumab PET Imaging and HER2 Specificity of Brain Metastases in HER2-Positive Breast Cancer Patients. EJNMMI Res. 2015, 5 (1), 8 10.1186/s13550-015-0082-6. 25853014 PMC4385241

[ref96] Reubi J. C.; Wenger S.; Schmuckli-Maurer J.; Schaer J.-C.; Gugger M. Bombesin Receptor Subtypes in Human Cancers: Detection with the Universal Radioligand (125)I-[D-TYR(6), Beta-ALA(11), PHE(13), NLE(14)] Bombesin(6–14). Clin. Cancer Res. 2002, 8 (4), 1139–1146. 11948125

[ref97] Reubi J.; Gugger M.; Waser B. Co-Expressed Peptide Receptors in Breast Cancer as a Molecular Basis for in Vivo Multireceptor Tumour Targeting. Eur. J. Nucl. Med. Mol. Imaging 2002, 29 (7), 855–862. 10.1007/s00259-002-0794-5. 12111125

[ref98] Gugger M.; Reubi J. C. Gastrin-Releasing Peptide Receptors in Non-Neoplastic and Neoplastic Human Breast. Am. J. Pathol. 1999, 155 (6), 2067–2076. 10.1016/S0002-9440(10)65525-3. 10595936 PMC1866930

[ref99] Dalm S. U.; Martens J. W. M.; Sieuwerts A. M.; van Deurzen C. H. M.; Koelewijn S. J.; de Blois E.; Maina T.; Nock B. A.; Brunel L.; Fehrentz J.-A.; Martinez J.; de Jong M.; Melis M. In Vitro and In Vivo Application of Radiolabeled Gastrin-Releasing Peptide Receptor Ligands in Breast Cancer. J. Nucl. Med. 2015, 56 (5), 752–757. 10.2967/jnumed.114.153023. 25791989

[ref100] Zhang K.; Aruva M. R.; Shanthly N.; Cardi C. A.; Patel C. A.; Rattan S.; Cesarone G.; Wickstrom E.; Thakur M. L. Vasoactive Intestinal Peptide (VIP) and Pituitary Adenylate Cyclase Activating Peptide (PACAP) Receptor Specific Peptide Analogues for PET Imaging of Breast Cancer: In Vitro/in Vivo Evaluation. Regul. Pept. 2007, 144 (1–3), 91–100. 10.1016/j.regpep.2007.06.008. 17727979 PMC2587158

[ref101] Reubi J. C. In Vitro Identification of Vasoactive Intestinal Peptide Receptors in Human Tumors: Implications for Tumor Imaging. J. Nucl. Med. 1995, 36 (10), 1846–1853. 7562054

[ref102] Maina T.; Bergsma H.; Kulkarni H. R.; Mueller D.; Charalambidis D.; Krenning E. P.; Nock B. A.; de Jong M.; Baum R. P. Preclinical and First Clinical Experience with the Gastrin-Releasing Peptide Receptor-Antagonist [68Ga]SB3 and PET/CT. Eur. J. Nucl. Med. Mol. Imaging 2016, 43 (5), 964–973. 10.1007/s00259-015-3232-1. 26631238

[ref103] Stoykow C.; Erbes T.; Maecke H. R.; Bulla S.; Bartholomä M.; Mayer S.; Drendel V.; Bronsert P.; Werner M.; Gitsch G.; Weber W. A.; Stickeler E.; Meyer P. T. Gastrin-Releasing Peptide Receptor Imaging in Breast Cancer Using the Receptor Antagonist ^68^ Ga-RM2 And PET. Theranostics 2016, 6 (10), 1641–1650. 10.7150/thno.14958. 27446498 PMC4955063

[ref104] Thakur M. L.; Zhang K.; Berger A.; Cavanaugh B.; Kim S.; Channappa C.; Frangos A. J.; Wickstrom E.; Intenzo C. M. VPAC1 Receptors for Imaging Breast Cancer: A Feasibility Study. J. Nucl. Med. 2013, 54 (7), 1019–1025. 10.2967/jnumed.112.114876. 23651947 PMC5506835

[ref105] Gusterson B.; Eaves C. J. Basal-like Breast Cancers: From Pathology to Biology and Back Again. Stem Cell Rep. 2018, 10 (6), 1676–1686. 10.1016/j.stemcr.2018.04.023. PMC6117459 29874626

[ref106] Lu J.; Steeg P. S.; Price J. E.; Krishnamurthy S.; Mani S. A.; Reuben J.; Cristofanilli M.; Dontu G.; Bidaut L.; Valero V.; Hortobagyi G. N.; Yu D. Breast Cancer Metastasis: Challenges and Opportunities. Cancer Res. 2009, 69 (12), 4951–4953. 10.1158/0008-5472.CAN-09-0099. 19470768

[ref107] Liu H.; Gruber C. W.; Alewood P. F.; Möller A.; Muttenthaler M. The Oxytocin Receptor Signalling System and Breast Cancer: A Critical Review. Oncogene 2020, 39 (37), 5917–5932. 10.1038/s41388-020-01415-8. 32782397 PMC7483001

[ref108] Lerman B.; Harricharran T.; Ogunwobi O. O. Oxytocin and Cancer: An Emerging Link. World J. Clin. Oncol. 2018, 9 (5), 74–82. 10.5306/wjco.v9.i5.74. 30254962 PMC6153127

[ref109] Jurek B.; Neumann I. D. The Oxytocin Receptor: From Intracellular Signaling to Behavior. Physiol. Rev. 2018, 98 (3), 1805–1908. 10.1152/physrev.00031.2017. 29897293

[ref110] Waltenspühl Y.; Schöppe J.; Ehrenmann J.; Kummer L.; Plückthun A. Crystal Structure of the Human Oxytocin Receptor. Sci. Adv. 2020, 6 (29), eabb5419 10.1126/sciadv.abb5419. 32832646 PMC7439316

[ref111] Meyerowitz J. G.; Robertson M. J.; Barros-Álvarez X.; Panova O.; Nwokonko R. M.; Gao Y.; Skiniotis G. The Oxytocin Signaling Complex Reveals a Molecular Switch for Cation Dependence. Nat. Struct. Mol. Biol. 2022, 29 (3), 274–281. 10.1038/s41594-022-00728-4. 35241813 PMC12101619

[ref112] Waltenspühl Y.; Ehrenmann J.; Vacca S.; Thom C.; Medalia O.; Plückthun A. Structural Basis for the Activation and Ligand Recognition of the Human Oxytocin Receptor. Nat. Commun. 2022, 13 (1), 4153 10.1038/s41467-022-31325-0. 35851571 PMC9293896

[ref113] Leveque T. F.; Scharrer E. Pituicytes and the Origin of the Antidiuretic Hormone ^1^. Endocrinology 1953, 52 (4), 436–447. 10.1210/endo-52-4-436. 13043556

[ref114] Neville M. C.; McFadden T. B.; Forsyth I. Hormonal Regulation of Mammary Differentiation and Milk Secretion. J. Mammary Gland Biol. Neoplasia 2002, 7 (1), 49–66. 10.1023/A:1015770423167. 12160086

[ref115] Cassoni P.; Sapino A.; Papotti M.; Bussolati G. Oxytocin and Oxytocin-Analogue F314 Inhibit Cell Proliferation and Tumor Growth of Rat and Mouse Mammary Carcinomas. Int. J. Cancer 1996, 66 (6), 817–820. 10.1002/(SICI)1097-0215(19960611)66:6<817::AID-IJC18>3.0.CO;2-#. 8647655

[ref116] Lee K. H.; Khan-Dawood F. S.; Dawood M. Y. Oxytocin Receptor and Its Messenger Ribonucleic Acid in Human Leiomyoma and Myometrium. Am. J. Obstet. Gynecol. 1998, 179 (3), 620–627. 10.1016/S0002-9378(98)70054-7. 9757961

[ref117] Cassoni P.; Sapino A.; Stella A.; Fortunati N.; Bussolati G. Presence and Significance of Oxytocin Receptors in Human Neuroblastomas and Glial Tumors. Int. J. Cancer 1998, 77 (5), 695–700. 10.1002/(SICI)1097-0215(19980831)77:5<695::AID-IJC6>3.0.CO;2-Q. 9688301

[ref118] Cassoni P.; Fulcheri E.; Carcangiu M. L.; Stella A.; Deaglio S.; Bussolati G. Oxytocin Receptors in Human Adenocarcinomas of the Endometrium: Presence and Biological Significance. J. Pathol. 2000, 190 (4), 470–477. 10.1002/(SICI)1096-9896(200003)190:4<470::AID-PATH550>3.0.CO;2-G. 10699997

[ref119] Morita T.; Shibata K.; Kikkawa F.; Kajiyama H.; Ino K.; Mizutani S. Oxytocin Inhibits the Progression of Human Ovarian Carcinoma Cellsin Vitro Andin Vivo. Int. J. Cancer 2004, 109 (4), 525–532. 10.1002/ijc.20017. 14991573

[ref120] Xu H.; Fu S.; Chen Q.; Gu M.; Zhou J.; Liu C.; Chen Y.; Wang Z. The Function of Oxytocin: A Potential Biomarker for Prostate Cancer Diagnosis and Promoter of Prostate Cancer. Oncotarget 2017, 8 (19), 31215–31226. 10.18632/oncotarget.16107. 28415720 PMC5458202

[ref121] Whittington K.; Assinder S.; Gould M.; Nicholson H. Oxytocin, Oxytocin-Associated Neurophysin and the Oxytocin Receptor in the Human Prostate. Cell Tissue Res. 2004, 318 (2), 375–382. 10.1007/s00441-004-0968-5. 15459766

[ref122] Whittington K.; Connors B.; King K.; Assinder S.; Hogarth K.; Nicholson H. The Effect of Oxytocin on Cell Proliferation in the Human Prostate Is Modulated by Gonadal Steroids: Implications for Benign Prostatic Hyperplasia and Carcinoma of the Prostate. Prostate 2007, 67 (10), 1132–1142. 10.1002/pros.20612. 17492653

[ref123] Péqueux C.; Keegan B. P.; Hagelstein M.-T.; Geenen V.; Legros J.-J.; North W. G. Oxytocin- and Vasopressin-Induced Growth of Human Small-Cell Lung Cancer Is Mediated by the Mitogen-Activated Protein Kinase Pathway. Endocr. Relat. Cancer 2004, 11 (4), 871–885. 10.1677/erc.1.00803. 15613460

[ref124] Cassoni P.; Sapino A.; Munaron L.; Deaglio S.; Chini B.; Graziani A.; Ahmed A.; Bussolati G. Activation of Functional Oxytocin Receptors Stimulates Cell Proliferation in Human Trophoblast and Choriocarcinoma Cell Lines*. Endocrinology 2001, 142 (3), 1130–1136. 10.1210/endo.142.3.8047. 11181528

[ref125] Petersson M. Opposite Effects of Oxytocin on Proliferation of Osteosarcoma Cell Lines. Regul. Pept. 2008, 150 (1–3), 50–54. 10.1016/j.regpep.2008.02.007. 18384894

[ref126] Petersson M.; Lagumdzija A.; Stark A.; Bucht E. Oxytocin Stimulates Proliferation of Human Osteoblast-like Cells. Peptides 2002, 23 (6), 1121–1126. 10.1016/S0196-9781(02)00041-4. 12126740

[ref127] Chini B.; Chinol M.; Cassoni P.; Papi S.; Reversi A.; Areces L.; Marrocco T.; Paganelli G.; Manning M.; Bussolati G. Improved Radiotracing of Oxytocin Receptor-Expressing Tumours Using the New [111In]-DOTA-Lys8-Deamino-Vasotocin Analogue. Br. J. Cancer 2003, 89 (5), 930–936. 10.1038/sj.bjc.6601189. 12942128 PMC2394487

[ref128] Miranda-Olvera A. D.; Ferro-Flores G.; Pedraza-López M.; de Murphy C. A.; De León-Rodríguez L. M. Synthesis of Oxytocin HYNIC Derivatives as Potential Diagnostic Agents for Breast Cancer. Bioconjugate Chem. 2007, 18 (5), 1560–1567. 10.1021/bc070047a. 17665873

[ref129] Cassoni P.; Marrocco T.; Sapino A.; Allia E.; Bussolati G. Oxytocin Synthesis within the Normal and Neoplastic Breast: First Evidence of a Local Peptide Source. Int. J. Oncol. 2006, 28 (5), 1263–1268. 10.3892/ijo.28.5.1263. 16596243

[ref130] Ito Y.; Kobayashi T.; Kimura T.; Matsuura N.; Wakasugi E.; Takeda T.; Shimano T.; Kubota Y.; Nobunaga T.; Makino Y.; Azuma C.; Saji F.; Monden M. Investigation of the Oxytocin Receptor Expression in Human Breast Cancer Tissue Using Newly Established Monoclonal Antibodies. Endocrinology 1996, 137 (2), 773–779. 10.1210/endo.137.2.8593829. 8593829

[ref131] Bussolati G.; Cassoni P.; Ghisolfi G.; Negro F.; Sapino A. Immunolocalization and Gene Expression of Oxytocin Receptors in Carcinomas and Non-Neoplastic Tissues of the Breast. Am. J. Pathol. 1996, 148 (6), 1895–1903. 8669475 PMC1861656

[ref132] Amico J. A.; Rauk P. N.; Cai H. Estradiol and Progesterone Regulate Oxytocin Receptor Binding and Expression in Human Breast Cancer Cell Lines. Endocrine 2002, 18 (1), 79–84. 10.1385/ENDO:18:1:79. 12166628

[ref133] Copland J. A.; Jeng Y.-J.; Strakova Z.; Ives K. L.; Hellmich M. R.; Soloff M. S. Demonstration of Functional Oxytocin Receptors in Human Breast Hs578T Cells and Their Up-Regulation through a Protein Kinase C-Dependent Pathway*. Endocrinology 1999, 140 (5), 2258–2267. 10.1210/endo.140.5.6723. 10218979

[ref134] Cassoni P.; Marrocco T.; Bussolati B.; Allia E.; Munaron L.; Sapino A.; Bussolati G. Oxytocin Induces Proliferation and Migration in Immortalized Human Dermal Microvascular Endothelial Cells and Human Breast Tumor-Derived Endothelial Cells. Mol. Cancer Res. 2006, 4 (6), 351–359. 10.1158/1541-7786.MCR-06-0024. 16778082

[ref135] Cassoni P.; Sapino A.; Fortunati N.; Munaron L.; Chini B.; Bussolati G. Oxytocin Inhibits the Proliferation of MDA-MB231 Human Breast-Cancer Cells via Cyclic Adenosine Monophosphate and Protein Kinase A. Int. J. Cancer 1997, 72 (2), 340–344. 10.1002/(SICI)1097-0215(19970717)72:2<340::AID-IJC23>3.0.CO;2-I. 9219843

[ref136] Cassoni P.; Sapino A.; Negro F.; Bussolati G. Oxytocin Inhibits Proliferation of Human Breast Cancer Cell Lines. Virchows Arch 1994, 425 (5), 467–472. 10.1007/BF00197549. 7850070

[ref137] Khori V.; Alizadeh A. M.; Khalighfard S.; Heidarian Y.; Khodayari H. Oxytocin Effects on the Inhibition of the NF-κB/miR195 Pathway in Mice Breast Cancer. Peptides 2018, 107, 54–60. 10.1016/j.peptides.2018.07.007. 30076862

[ref138] Alizadeh A. M.; Heydari Z.; Rahimi M.; Bazgir B.; Shirvani H.; Alipour S.; Heidarian Y.; Khalighfard S.; Isanejad A. Oxytocin Mediates the Beneficial Effects of the Exercise Training on Breast Cancer. Exp. Physiol. 2018, 103 (2), 222–235. 10.1113/EP086463. 29143998

[ref139] Sapino A.; Cassoni P.; Stella A.; Bussolati G. Oxytocin Receptor within the Breast: Biological Function and Distribution. Anticancer Res. 1998, 18 (3C), 2181–2186. 9703781

[ref140] Reversi A.; Rimoldi V.; Marrocco T.; Cassoni P.; Bussolati G.; Parenti M.; Chini B. The Oxytocin Receptor Antagonist Atosiban Inhibits Cell Growth via a “Biased Agonist” Mechanism. J. Biol. Chem. 2005, 280 (16), 16311–16318. 10.1074/jbc.M409945200. 15705593

[ref141] Imanieh M. H.; Bagheri F.; Alizadeh A. M.; Ashkani-Esfahani S. Oxytocin Has Therapeutic Effects on Cancer, a Hypothesis. Eur. J. Pharmacol. 2014, 741, 112–123. 10.1016/j.ejphar.2014.07.053. 25094035

[ref142] Lee A. V.; Oesterreich S.; Davidson N. E. MCF-7 Cells-Changing the Course of Breast Cancer Research and Care for 45 Years. JNCI J. Natl. Cancer Inst. 2015, 107 (7), djv073–djv073. 10.1093/jnci/djv073. 25828948

[ref143] De Giovanni; Nicoletti; Landuzzi; Palladini; Lollini; Nanni Bioprofiling TS/A Murine Mammary Cancer for a Functional Precision Experimental Model. Cancers 2019, 11 (12), 1889 10.3390/cancers11121889. 31783695 PMC6966465

[ref144] Lanari C.; Lüthy I.; Lamb C. A.; Fabris V.; Pagano E.; Helguero L. A.; Sanjuan N.; Merani S.; Molinolo A. A. Five Novel Hormone-Responsive Cell Lines Derived from Murine Mammary Ductal Carcinomas: In Vivo and in Vitro Effects of Estrogens and Progestins. Cancer Res. 2001, 61 (1), 293–302. 11196177

[ref145] Ariana M.; Pornour M.; Mehr S. S.; Vaseghi H.; Ganji S. M.; Alivand M. R.; Salari M.; Akbari M. E. Preventive Effects of Oxytocin and Oxytocin Receptor in Breast Cancer Pathogenesis. Pers. Med. 2019, 16 (1), 25–34. 10.2217/pme-2018-0009. 30451597

[ref146] Lewison E. F. Breast Cancer and Pregnancy or Lactation. Int. Abstr. Surg. 1954, 99 (5), 417–424. 13205440

[ref147] Lyons T. R.; Schedin P. J.; Borges V. F. Pregnancy and Breast Cancer: When They Collide. J. Mammary Gland Biol. Neoplasia 2009, 14 (2), 87–98. 10.1007/s10911-009-9119-7. 19381788 PMC2693784

[ref148] Breast Cancer and Breastfeeding: Collaborative Reanalysis of Individual Data from 47 Epidemiological Studies in 30 Countries, Including 50 302 Women with Breast Cancer and 96 973 Women without the Disease. Lancet 2002, 360 (9328), 187–195. 10.1016/S0140-6736(02)09454-0. 12133652

[ref149] Lipworth L. History of Breast-Feeding in Relation to Breast Cancer Risk: A Review of the Epidemiologic Literature. J. Natl. Cancer Inst. 2000, 92 (4), 302–312. 10.1093/jnci/92.4.302. 10675379

[ref150] Yang L.; Jacobsen K. H. A Systematic Review of the Association between Breastfeeding and Breast Cancer. J. Womens Health 2008, 17 (10), 1635–1645. 10.1089/jwh.2008.0917. 19049358

[ref151] Kotsopoulos J.; Lubinski J.; Salmena L.; Lynch H. T.; Kim-Sing C.; Foulkes W. D.; Ghadirian P.; Neuhausen S. L.; Demsky R.; Tung N.; Ainsworth P.; Senter L.; Eisen A.; Eng C.; Singer C.; Ginsburg O.; Blum J.; Huzarski T.; Poll A.; Sun P.; Narod S. A. Hereditary Breast Cancer Clinical Study Group. Breastfeeding and the Risk of Breast Cancer in BRCA1 and BRCA2Mutation Carriers. Breast Cancer Res. BCR 2012, 14 (2), R42 10.1186/bcr3138. 22405187 PMC3446376

[ref152] Hinkula M.; Pukkala E.; Kyyrönen P.; Kauppila A. Grand Multiparity and the Risk of Breast Cancer: Population-Based Study in Finland. Cancer Causes Control. 2001, 12 (6), 491–500. 10.1023/A:1011253527605. 11519757

[ref153] Lee S. Y.; Kim M. T.; Kim S. W.; Song M. S.; Yoon S. J. Effect of Lifetime Lactation on Breast Cancer Risk: A Korean Women’s Cohort Study. Int. J. Cancer 2003, 105 (3), 390–393. 10.1002/ijc.11078. 12704674

[ref154] Shema L.; Ore L.; Ben-Shachar M.; Haj M.; Linn S. The Association between Breastfeeding and Breast Cancer Occurrence among Israeli Jewish Women: A Case Control Study. J. Cancer Res. Clin. Oncol. 2007, 133 (8), 539 10.1007/s00432-007-0199-8. 17453241 PMC12160900

[ref155] De Silva M.; Senarath U.; Gunatilake M.; Lokuhetty D. Prolonged Breastfeeding Reduces Risk of Breast Cancer in Sri Lankan Women: A Case-Control Study. Cancer Epidemiol. 2010, 34 (3), 267–273. 10.1016/j.canep.2010.02.012. 20338838

[ref156] Bussolati G.; Chinol M.; Chini B.; Nacca A.; Cassoni P.; Paganelli G. 111In-Labeled 1,4,7,10-Tetraazacyclododecane-N,N’,N″,N″’-Tetraacetic Acid-Lys(8)-Vasotocin: A New Powerful Radioligand for Oxytocin Receptor-Expressing Tumors. Cancer Res. 2001, 61 (11), 4393–4397. 11389066

[ref157] Gruber C. W.; Muttenthaler M.; Freissmuth M. Ligand-Based Peptide Design and Combinatorial Peptide Libraries to Target G Protein-Coupled Receptors. Curr. Pharm. Des. 2010, 16 (28), 3071–3088. 10.2174/138161210793292474. 20687879 PMC4939874

[ref158] Manning M.; Misicka A.; Olma A.; Bankowski K.; Stoev S.; Chini B.; Durroux T.; Mouillac B.; Corbani M.; Guillon G. Oxytocin and Vasopressin Agonists and Antagonists as Research Tools and Potential Therapeutics. J. Neuroendocrinol. 2012, 24 (4), 609–628. 10.1111/j.1365-2826.2012.02303.x. 22375852 PMC3490377

[ref159] Gimpl G.; Fahrenholz F. The Oxytocin Receptor System: Structure, Function, and Regulation. Physiol. Rev. 2001, 81 (2), 629–683. 10.1152/physrev.2001.81.2.629. 11274341

[ref160] Wiśniewski K.; Alagarsamy S.; Galyean R.; Tariga H.; Thompson D.; Ly B.; Wiśniewska H.; Qi S.; Croston G.; Laporte R.; Rivière P. J.-M.; Schteingart C. D. New, Potent, and Selective Peptidic Oxytocin Receptor Agonists. J. Med. Chem. 2014, 57 (12), 5306–5317. 10.1021/jm500365s. 24874785

[ref161] Muttenthaler M.; Andersson Å.; Vetter I.; Menon R.; Busnelli M.; Ragnarsson L.; Bergmayr C.; Arrowsmith S.; Deuis J. R.; Chiu H. S.; Palpant N. J.; O’Brien M.; Smith T. J.; Wray S.; Neumann I. D.; Gruber C. W.; Lewis R. J.; Alewood P. F. Subtle Modifications to Oxytocin Produce Ligands That Retain Potency and Improved Selectivity across Species. Sci. Signal. 2017, 10 (508), eaan3398 10.1126/scisignal.aan3398. 29208680 PMC5892705

[ref162] Koehbach J.; O’Brien M.; Muttenthaler M.; Miazzo M.; Akcan M.; Elliott A. G.; Daly N. L.; Harvey P. J.; Arrowsmith S.; Gunasekera S.; Smith T. J.; Wray S.; Göransson U.; Dawson P. E.; Craik D. J.; Freissmuth M.; Gruber C. W. Oxytocic Plant Cyclotides as Templates for Peptide G Protein-Coupled Receptor Ligand Design. Proc. Natl. Acad. Sci. U. S. A. 2013, 110 (52), 21183–21188. 10.1073/pnas.1311183110. 24248349 PMC3876230

[ref163] Beard R.; Stucki A.; Schmitt M.; Py G.; Grundschober C.; Gee A. D.; Tate E. W. Building Bridges for Highly Selective, Potent and Stable Oxytocin and Vasopressin Analogs. Bioorg. Med. Chem. 2018, 26 (11), 3039–3045. 10.1016/j.bmc.2018.03.019. 29602673

[ref164] Adachi Y.; Sakimura K.; Shimizu Y.; Nakayama M.; Terao Y.; Yano T.; Asami T. Potent and Selective Oxytocin Receptor Agonists without Disulfide Bridges. Bioorg. Med. Chem. Lett. 2017, 27 (11), 2331–2335. 10.1016/j.bmcl.2017.04.030. 28438540

[ref165] Alnouri M. W.; Jepards S.; Casari A.; Schiedel A. C.; Hinz S.; Müller C. E. Selectivity Is Species-Dependent: Characterization of Standard Agonists and Antagonists at Human, Rat, and Mouse Adenosine Receptors. Purinergic Signal. 2015, 11 (3), 389–407. 10.1007/s11302-015-9460-9. 26126429 PMC4529847

[ref166] Postina R.; Kojro E.; Fahrenholz F. Identification of Neurohypophysial Hormone Receptor Domains Involved in Ligand Binding and G Protein Coupling. In Vasopressin and Oxytocin; Zingg H. H., Bourque C. W., Bichet D. G., Eds.; Advances in Experimental Medicine and Biology 449; Springer US: Boston, MA, 1998; pp 371–385. 10.1007/978-1-4615-4871-3_48. 10026828

[ref167] Fanelli F.; Barbier P.; Zanchetta D.; de Benedetti P. G.; Chini B. Activation Mechanism of Human Oxytocin Receptor: A Combined Study of Experimental and Computer-Simulated Mutagenesis. Mol. Pharmacol. 1999, 56 (1), 214–225. 10.1124/mol.56.1.214. 10385703

[ref168] Favre N.; Fanelli F.; Missotten M.; Nichols A.; Wilson J.; di Tiani M.; Rommel C.; Scheer A. The DRY Motif as a Molecular Switch of the Human Oxytocin Receptor. Biochemistry 2005, 44 (30), 9990–10008. 10.1021/bi0509853. 16042376

[ref169] Terrillon S.; Cheng L. L.; Stoev S.; Mouillac B.; Barberis C.; Manning M.; Durroux T. Synthesis and Characterization of Fluorescent Antagonists and Agonists for Human Oxytocin and Vasopressin V _1 a_ Receptors. J. Med. Chem. 2002, 45 (12), 2579–2588. 10.1021/jm010526+. 12036367

[ref170] Corbani M.; Trueba M.; Stoev S.; Murat B.; Mion J.; Boulay V.; Guillon G.; Manning M. Design, Synthesis, and Pharmacological Characterization of Fluorescent Peptides for Imaging Human V1b Vasopressin or Oxytocin Receptors. J. Med. Chem. 2011, 54 (8), 2864–2877. 10.1021/jm1016208. 21428295 PMC3104497

[ref171] Mouillac B.; Manning M.; Durroux T. Fluorescent Agonists and Antagonists for Vasopressin/Oxytocin G Protein-Coupled Receptors: Usefulness in Ligand Screening Assays and Receptor Studies. Mini-Rev. Med. Chem. 2008, 8 (10), 996–1005. 10.2174/138955708785740607. 18782052 PMC2763091

[ref172] Albizu L.; Teppaz G.; Seyer R.; Bazin H.; Ansanay H.; Manning M.; Mouillac B.; Durroux T. Toward Efficient Drug Screening by Homogeneous Assays Based on the Development of New Fluorescent Vasopressin and Oxytocin Receptor Ligands. J. Med. Chem. 2007, 50 (20), 4976–4985. 10.1021/jm061404q. 17850055

[ref173] Hope D. B.; Murti V. V.; Du Vigneaud V. A Highly Potent Analogue of Oxytocin, Desamino-Oxytocin. J. Biol. Chem. 1962, 237, 1563–1566. 10.1016/S0021-9258(19)83740-7. 14448781

[ref174] Manning M.; Miteva K.; Pancheva S.; Stoev S.; Wo N. C.; Chan W. Y. Design and Synthesis of Highly Selective in Vitro and in Vivo Uterine Receptor Antagonists of Oxytocin: Comparisons with Atosiban. Int. J. Pept. Protein Res. 1995, 46 (3–4), 244–252. 10.1111/j.1399-3011.1995.tb00596.x. 8537178

[ref175] Chini B.; Mouillac B.; Ala Y.; Balestre M. N.; Trumpp-Kallmeyer S.; Hoflack J.; Elands J.; Hibert M.; Manning M.; Jard S. Tyr115 Is the Key Residue for Determining Agonist Selectivity in the V1a Vasopressin Receptor. EMBO J. 1995, 14 (10), 2176–2182. 10.1002/j.1460-2075.1995.tb07211.x. 7774575 PMC398323

[ref176] Busnelli M.; Saulière A.; Manning M.; Bouvier M.; Galés C.; Chini B. Functional Selective Oxytocin-Derived Agonists Discriminate between Individual G Protein Family Subtypes. J. Biol. Chem. 2012, 287 (6), 3617–3629. 10.1074/jbc.M111.277178. 22069312 PMC3281696

[ref177] Lowbridge J.; Manning M.; Haldar J.; Sawyer W. H. Synthesis and Some Pharmacological Properties of [4-Threonine,7-Glycine]Oxytocin, [1-(L-2-Hydroxy-3-Mercaptopropanoic Acid),4-Threonine,7-Glycine]Oxytocin (Hydroxy[Thr4, Gly7]Oxytocin), and [7-Glycine]Oxytocin, Peptides with High Oxytocic-Antidiuretic Selectivity. J. Med. Chem. 1977, 20 (1), 120–123. 10.1021/jm00211a025. 833810

[ref178] Busnelli M.; Kleinau G.; Muttenthaler M.; Stoev S.; Manning M.; Bibic L.; Howell L. A.; McCormick P. J.; Di Lascio S.; Braida D.; Sala M.; Rovati G. E.; Bellini T.; Chini B. Design and Characterization of Superpotent Bivalent Ligands Targeting Oxytocin Receptor Dimers via a Channel-Like Structure. J. Med. Chem. 2016, 59 (15), 7152–7166. 10.1021/acs.jmedchem.6b00564. 27420737

[ref179] Jelinski M.; Hamacher K.; Coenen H. H. C-Terminal18F-Fluoroethylamidation Exemplified on [Gly-OH9] Oxytocin. J. Label. Compd. Radiopharm. 2002, 45 (3), 217–229. 10.1002/jlcr.547.

[ref180] Beard R.; Singh N.; Grundschober C.; Gee A. D.; Tate E. W. High-Yielding ^18^ F Radiosynthesis of a Novel Oxytocin Receptor Tracer, a Probe for Nose-to-Brain Oxytocin Uptake *in Vivo*. Chem. Commun. 2018, 54 (58), 8120–8123. 10.1039/C8CC01400K. PMC6049614 29974895

[ref181] Monoclonal Antibody and Peptide-Targeted Radiotherapy of Cancer; Reilly R., Ed.; Wiley: Hoboken, NJ, 2010.

[ref182] Liu S.; Edwards D. S.; Looby R. J.; Harris A. R.; Poirier M. J.; Barrett J. A.; Heminway S. J.; Carroll T. R. Labeling a Hydrazino Nicotinamide-Modified Cyclic IIb/IIIa Receptor Antagonist with 99mTc Using Aminocarboxylates as Coligands. Bioconjugate Chem. 1996, 7 (1), 63–71. 10.1021/bc950069+. 8741992

[ref183] Abrams M. J.; Juweid M.; tenKate C. I.; Schwartz D. A.; Hauser M. M.; Gaul F. E.; Fuccello A. J.; Rubin R. H.; Strauss H. W.; Fischman A. J. Technetium-99m-Human Polyclonal IgG Radiolabeled via the Hydrazino Nicotinamide Derivative for Imaging Focal Sites of Infection in Rats. J. Nucl. Med. 1990, 31 (12), 2022–2028. 2266401

[ref184] Zingg H. H.; Laporte S. A. The Oxytocin Receptor. Trends Endocrinol. Metab. 2003, 14 (5), 222–227. 10.1016/S1043-2760(03)00080-8. 12826328

[ref185] Chini B.; Mouillac B.; Balestre M.-N.; Trumpp-Kallmeyer S.; Hoflack J.; Hibert M.; Andriolo M.; Pupier S.; Jard S.; Barberis C. Two Aromatic Residues Regulate the Response of the Human Oxytocin Receptor to the Partial Agonist Arginine Vasopressin. FEBS Lett. 1996, 397 (2–3), 201–206. 10.1016/S0014-5793(96)01135-0. 8955347

[ref186] Harding F. A.; Stickler M. M.; Razo J.; DuBridge R. The Immunogenicity of Humanized and Fully Human Antibodies: Residual Immunogenicity Resides in the CDR Regions. mAbs 2010, 2 (3), 256–265. 10.4161/mabs.2.3.11641. 20400861 PMC2881252

[ref187] Abbasi Gharibkandi N.; Conlon J. M.; Hosseinimehr S. J. Strategies for Improving Stability and Pharmacokinetic Characteristics of Radiolabeled Peptides for Imaging and Therapy. Peptides 2020, 133, 170385 10.1016/j.peptides.2020.170385. 32822772

[ref188] AlDeghaither D.; Smaglo B. G.; Weiner L. M. Beyond Peptides and mAbs-Current Status and Future Perspectives for Biotherapeutics with Novel Constructs. J. Clin. Pharmacol. 2015, 55 (S3), S4–S20. 10.1002/jcph.407. 25707963 PMC4340091

[ref189] Bodei L.; Cremonesi M.; Zoboli S.; Grana C.; Bartolomei M.; Rocca P.; Caracciolo M.; Mäcke H. R.; Chinol M.; Paganelli G. Receptor-Mediated Radionuclide Therapy with 90Y-DOTATOC in Association with Amino Acid Infusion: A Phase I Study. Eur. J. Nucl. Med. Mol. Imaging 2003, 30 (2), 207–216. 10.1007/s00259-002-1023-y. 12552338

[ref190] Sofou S. Radionuclide Carriers for Targeting of Cancer. Int. J. Nanomedicine 2008, 181 10.2147/IJN.S2736. 18686778 PMC2527672

[ref191] Urruticoechea A.; Alemany R.; Balart J.; Villanueva A.; Vinals F.; Capella G. Recent Advances in Cancer Therapy: An Overview. Curr. Pharm. Des. 2010, 16 (1), 3–10. 10.2174/138161210789941847. 20214614

[ref192] Feijtel D.; de Jong M.; Nonnekens J. Peptide Receptor Radionuclide Therapy: Looking Back, Looking Forward. Curr. Top. Med. Chem. 2020, 20 (32), 2959–2969. 10.2174/1568026620666200226104652. 32101125 PMC8493789

[ref193] Opalińska M.; Sowa-Staszczak A.; Grochowska A.; Olearska H.; Hubalewska-Dydejczyk A. Value of Peptide Receptor Radionuclide Therapy as Neoadjuvant Treatment in the Management of Primary Inoperable Neuroendocrine Tumors. Front. Oncol. 2021, 11, 687925 10.3389/fonc.2021.687925. 34868906 PMC8633407

[ref194] Camus B.; Cottereau A.-S.; Palmieri L.-J.; Dermine S.; Tenenbaum F.; Brezault C.; Coriat R. Indications of Peptide Receptor Radionuclide Therapy (PRRT) in Gastroenteropancreatic and Pulmonary Neuroendocrine Tumors: An Updated Review. J. Clin. Med. 2021, 10 (6), 1267 10.3390/jcm10061267. 33803817 PMC8003169

[ref195] Hennrich U.; Kopka K. Lutathera®: The First FDA- and EMA-Approved Radiopharmaceutical for Peptide Receptor Radionuclide Therapy. Pharmaceuticals 2019, 12 (3), 114 10.3390/ph12030114. 31362406 PMC6789871

[ref196] Sgouros G.; Bodei L.; McDevitt M. R.; Nedrow J. R. Radiopharmaceutical Therapy in Cancer: Clinical Advances and Challenges. Nat. Rev. Drug Discovery 2020, 19 (9), 589–608. 10.1038/s41573-020-0073-9. 32728208 PMC7390460

[ref197] Kręcisz P.; Czarnecka K.; Królicki L.; Mikiciuk-Olasik E.; Szymański P. Radiolabeled Peptides and Antibodies in Medicine. Bioconjugate Chem. 2021, 32 (1), 25–42. 10.1021/acs.bioconjchem.0c00617. PMC7872318 33325685

[ref198] Erdmann S.; Niederstadt L.; Koziolek E. J.; Gómez J. D. C.; Prasad S.; Wagener A.; von Hacht J. L.; Reinicke S.; Exner S.; Bandholtz S.; Beindorff N.; Brenner W.; Grötzinger C. CMKLR1-Targeting Peptide Tracers for PET/MR Imaging of Breast Cancer. Theranostics 2019, 9 (22), 6719–6733. 10.7150/thno.34857. 31588246 PMC6771245

[ref199] Nock B. A.; Kaloudi A.; Lymperis E.; Giarika A.; Kulkarni H. R.; Klette I.; Singh A.; Krenning E. P.; de Jong M.; Maina T.; Baum R. P. Theranostic Perspectives in Prostate Cancer with the Gastrin-Releasing Peptide Receptor Antagonist NeoBOMB1: Preclinical and First Clinical Results. J. Nucl. Med. 2017, 58 (1), 75–80. 10.2967/jnumed.116.178889. 27493272

[ref200] Djaileb L.; Morgat C.; van der Veldt A.; Virgolini I.; Cortes F.; Demange A.; Orlandi F.; Wegener A. Preliminary Diagnostic Performance of [68Ga]-NeoBOMB1 in Patients with Gastrin-Releasing Peptide Receptor-Positive Breast, Prostate, Colorectal or Lung Tumors (NeoFIND). J. Nucl. Med. 2020, 61, 346.

[ref201] Nhàn N. T. T.; Yamada T.; Yamada K. H. Peptide-Based Agents for Cancer Treatment: Current Applications and Future Directions. Int. J. Mol. Sci. 2023, 24 (16), 12931 10.3390/ijms241612931. 37629112 PMC10454368

[ref202] Barman P.; Joshi S.; Sharma S.; Preet S.; Sharma S.; Saini A. Strategic Approaches to Improvise Peptide Drugs as Next Generation Therapeutics. Int. J. Pept. Res. Ther. 2023, 29 (4), 61 10.1007/s10989-023-10524-3. 37251528 PMC10206374

